# Objective impairments of gait and balance in adults living with HIV-1 infection: a systematic review and meta-analysis of observational studies

**DOI:** 10.1186/s12891-017-1682-2

**Published:** 2017-08-01

**Authors:** Karina Berner, Linzette Morris, Jochen Baumeister, Quinette Louw

**Affiliations:** 1 Division of Physiotherapy/Central Analytical Facilities (CAF) 3D Human Biomechanics Unit, Department of Rehabilitation & Health Sciences, Faculty of Medicine and Health Sciences, Stellenbosch University, PO Box 241, Cape Town, 8000 South Africa; 2 Exercise & Neuroscience Unit, Institute of Health, Nutrition and Sports Sciences, Europa-Universität Flensburg, Auf dem Campus 1, 24943 Flensburg, Germany

**Keywords:** HIV-1 infection, Gait, Postural balance, Falls

## Abstract

**Background:**

Gait and balance deficits are reported in adults with HIV infection and are associated with reduced quality of life. Current research suggests an increased fall-incidence in this population, with fall rates among middle-aged adults with HIV approximating that in seronegative elderly populations. Gait and postural balance rely on a complex interaction of the motor system, sensory control, and cognitive function. However, due to disease progression and complications related to ongoing inflammation, these systems may be compromised in people with HIV. Consequently, locomotor impairments may result that can contribute to higher-than-expected fall rates. The aim of this review was to synthesize the evidence regarding objective gait and balance impairments in adults with HIV, and to emphasize those which could contribute to increased fall risk.

**Methods:**

This review followed the Preferred Reporting Items for Systematic Reviews and Meta-Analyses (PRISMA) guidelines. An electronic search of published observational studies was conducted in March 2016. Methodological quality was assessed using the NIH Quality Assessment Tool for Observational Cohort and Cross-Sectional Studies. Narrative synthesis of gait and balance outcomes was performed, and meta-analyses where possible.

**Results:**

Seventeen studies were included, with fair to low methodological quality. All studies used clinical tests for gait-assessment. Gait outcomes assessed were speed, initiation-time and cadence. No studies assessed kinetics or kinematics. Balance was assessed using both instrumented and clinical tests. Outcomes were mainly related to center of pressure, postural reflex latencies, and timed clinical tests. There is some agreement that adults with HIV walk slower and have increased center of pressure excursions and -long loop postural reflex latencies, particularly under challenging conditions.

**Conclusions:**

Gait and balance impairments exist in people with HIV, resembling fall-associated parameters in the elderly. Impairments are more pronounced during challenging conditions, might be associated with disease severity, are not influenced by antiretroviral therapy, and might not be associated with peripheral neuropathy. Results should be interpreted cautiously due to overall poor methodological quality and heterogeneity. Locomotor impairments in adults with HIV are currently insufficiently quantified. Future research involving more methodological uniformity is warranted to better understand such impairments and to inform clinical decision-making, including fall-prevention strategies, in this population.

**Electronic supplementary material:**

The online version of this article (doi:10.1186/s12891-017-1682-2) contains supplementary material, which is available to authorized users.

## Background

In Southern Africa, about 18.9% of adults aged 15–49 years are HIV-1-seropositive [1]. Both globally and in Sub-Saharan Africa, life-expectancy in people living with HIV (PLHIV) is now comparable to that of seronegative adults [2–4]. HIV/AIDS has evolved into a chronic condition due to the success of highly-active antiretroviral therapy (HAART) [2], but this is paralleled by increasing morbidity. In 1990, HIV/AIDS was the 33rd most important cause of disability-adjusted life years (DALYs) globally, but has since increased to fifth position [5]. In Sub-Saharan Africa, a high prevalence of HIV-associated disability, including impairments in mobility and motor function, is reported in PLHIV [6].

Gait and balance deficits have been reported in PLHIV despite controlled viral load [7–10] and are associated with reduced quality of life (QOL) [11, 12]. Current HAART regimes have less neurotoxic effects than older versions, and thus there is a lower risk of developing peripheral neuropathy [13]. However, the prevalence of peripheral neuropathy remains quite high among PLHIV (between 30% and 62%), and the prevalence of locomotor impairments remains a concern [14–17]. PLHIV have an increased incidence of falls [18–20], and fall rates among middle-aged PLHIV are comparable to that of seronegative older adults, aged 65 years and older [18]. These falls are attributed to balance impairments [20].

HIV-1 infection may compromise motor function at multiple levels of the nervous system [13]. Structural MRI-studies have shown that PLHIV present with white matter alterations, including reduced pontocerebellar tract integrity, leading to gait and postural instability [21]. It remains unclear whether gait and balance impairments noted in PLHIV are due to the disease process or its treatment [22]. One hypothesis is that these deficits occur as a complication of ongoing inflammation [14, 23, 24]. PLHIV, although adherent to treatment, experience non-AIDS defining complications resembling geriatric processes at an earlier than expected age [23, 25]. Chronic immune activation may be an underlying mechanism [23]. This accelerated aging manifests in middle-aged PLHIV as the accumulation of various co-morbidities, including frailty [23, 26].

Of further concern is that PLHIV are four times more at risk of fractures due to accelerated bone demineralization [27] and sarcopenia [28], and the proposed interplay between these conditions [29]. Low bone mineral density and sarcopenia are associated with balance problems and falls [28, 30]. These complications may be intrinsic to HIV infection (e.g. due to metabolic changes) or HAART-induced [31]. It has been suggested that the loss in bone mineral density is a result of increased bone turnover, especially during the first 12 to 24 months after HAART-initiation [32–34]. Various protease inhibitor (PI) or nucleoside reverse transcriptase inhibitor (NRTI) type antiretroviral therapies (ART) show a correlation with mitochondrial toxicity [22], damaging the structure and function of muscles. In addition, reduced central activation of muscles has also been reported in PLHIV, likely due to impaired oxygen utilisation [35].

Information is building that PLHIV demonstrate gait and balance impairments. However, owing to the variety of observational data, it is difficult to quantify the extent of impairment and to gain insight into which parameters are truly affected and clinically relevant. In elderly populations, several gait and balance parameters have been identified as independent predictors of fall risk, including spatiotemporal, kinetic, kinematic and clinical [36–39]. To the authors’ knowledge, no previous systematic review has yet investigated objective impairments of gait and balance in PLHIV. The aim of this review is therefore to synthesize the evidence of objective impairments of gait and balance associated with HIV-1 infection, and to emphasize those which could contribute to increased fall risk. We also aimed to describe the evidence in relation to disease severity, treatment effects, task difficulty, and peripheral neuropathy.

## Methods

This systematic review was conducted according to the Preferred Reporting Items for Systematic Reviews and Meta-Analyses (PRISMA) guidelines [40].

### Criteria for considering studies for this review

Cohort, case-control and cross-sectional studies published in English as peer-reviewed journal articles were considered. Studies were included if they aimed to assess instrumented or non-instrumented objective parameters of gait and/or balance in adults (18–65 years of age) with HIV-1 infection, irrespective of gender. Given the expectation that there would be a paucity of information, studies with and without comparison groups were considered. Quantitative gait outcomes included, but were not limited to, kinematics, kinetics, spatiotemporal measures or clinical tests. Quantitative balance outcomes included, but were not limited to, biomechanical parameters such as center of pressure (COP) measures, and temporal measures via clinical tests. Studies were excluded if participants’ age exceeded 65 years, as the prevalence of locomotor impairments is known to increase in older age even in healthy populations [41]. Studies aiming to assess HIV-Associated Neurocognitive Disorder using a neuropsychological test battery were also excluded, regardless of the use of a gross motor component, in an attempt to focus on studies with the primary aim of objectively assessing and describing gait or balance in PLHIV.

### Search methods for identification of studies

#### Information sources

Six computerized bibliographic databases were searched, namely PubMed, Science Direct, EBSCOhost (CINAHL, MEDLINE, Africa-Wide Information), Scopus, ProQuest Medical Library and Google Scholar. Following a preliminary search of PubMed, a comprehensive search strategy, including all relevant key word/terms and medical subject headings (MeSH) was developed and adapted for use in subsequent searching of the remaining databases. Search terms included: (HIV-1 OR HIV Infection*) AND (motor function OR biomechanical phenomena OR gait OR postural balance OR locomotor function). The search was restricted to papers published from inception of the database to March 2016. Reference lists of all identified documents were hand-searched to identify additional relevant evidence. In the event of missing data, an attempt was made to contact the authors.

#### Study selection

Titles and abstracts of all initial hits were screened by one reviewer (KB). When necessary, consultation with a second reviewer (QL) was pursued. All potential full texts were subsequently screened by these two reviewers, and eligibility criteria were applied independently. Any discrepancies regarding eligibility were discussed between reviewers to reach consensus.

### Data collection and analysis

#### Methodological quality appraisal

One reviewer (KB) appraised the methodological quality of each included study using the National Institutes of Health (NIH) Quality Assessment Tool for Observational Cohort and Cross-Sectional Studies [42]. The tool is designed to aid appraisal of internal validity (potential risk of selection-, information-, or measurement bias, or confounding) of cross-sectional and cohort studies and was therefore appropriate for this review. It comprizes 14 criteria. All criteria can be answered as “yes”, “no”, “cannot determine”, “not applicable” or “not reported”. All responses other than “yes” indicate risk of bias. Inherent to the design, cross-sectional studies automatically score “not applicable” on criteria 6, 7, 10 and 13. After all 17 articles were scored by the first reviewer, two of these were randomly selected for audit and independently scored by a second reviewer (LM). The scores assigned by each reviewer were compared by specifically discussing those criteria with discrepant scores. Consistent discrepancies were noted specifically for criteria 6, 10 and 13 for both studies – which were resolved after agreeing that these criteria should be scored as “not applicable” as per the instrument’s instructions. Resultant total scores were similar; thus it was not deemed necessary for the second reviewer to score the remaining 15 articles as well. Each criterion was weighted equally in the overall grading, and studies were not excluded based on quality score, due to the expected dearth of information.

#### Data extraction

Data extracted from each study were summarized using a customized Excel spreadsheet, based on Cochrane forms. Information about sample demographics as well as the study aims, study design, known confounders to gait and balance, descriptors of HIV-disease, gait or balance analysis tool or test used, specific objective gait or balance outcomes, dose-response evidence, treatment effects, association of disease severity, association of peripheral neuropathy, findings and limitations of each study were extracted. Principle summary measures were means and standard deviations (SD).

#### Data analysis or synthesis

Narrative description of data was done using text summaries or tables as appropriate. For outcomes that were reported in at least two studies, a meta-analysis was conducted in Revman version 5.2, provided that homogeneity in the outcomes and samples existed regarding units of measurement, test conditions, gender and disease severity. Mean differences and 95% confidence intervals (CI) were calculated via a random effects model, provided that means and SD were reported, and were presented graphically as forest plots. Symptomatic (presenting with various symptoms of chronic HIV disease) and asymptomatic (asymptomatic HIV infection/clinically latent phase of HIV) subgroups of PLHIV were analyzed.

## Results

### Study selection

**Fig. 1:**
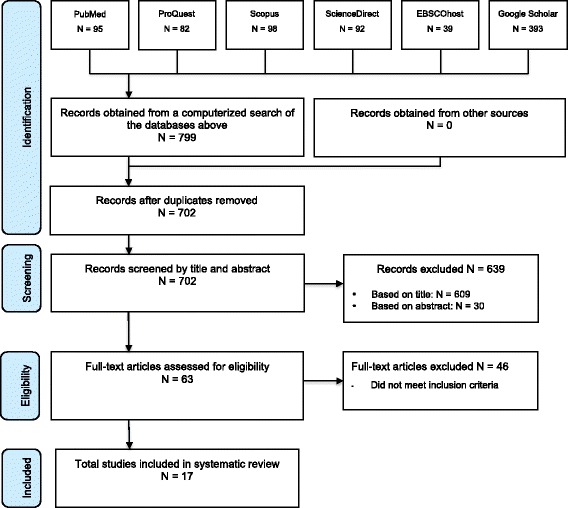
PRISMA flow diagram of literature search and selection process

The initial search in March 2016 produced 799 total hits (Fig. 1). After removing duplicates and applying eligibility criteria, 93 potential titles remained. Thirty studies were subsequently excluded upon reading the abstracts. The main reasons for exclusion were that the outcome measures were not relevant to the review question, participants were not within the specified age range, and study design was inappropriate. Following full text review, the number of studies for inclusion was reduced to 17. Primary reasons for exclusion were inability to obtain full text, ineligible participants, no raw data and outcomes that were not relevant to the review question.

### Study characteristics

#### Critical appraisal of study quality

Table 1 presents the methodological quality appraisal scores of the included studies, which ranged from fair to poor. A mean score of 40.34% was obtained, ranging from 7.14% (lowest internal validity amongst the included studies) to 57.14% (strongest internal validity amongst the included studies).

**Table 1:** Methodological quality appraisal

		Trenkwalder 1992 [46]	Arendt 1994 [47]	Beckley 1998 [50]	Bauer 2005 [7]	Dellepiane 2005 [48]	Simmonds 2005 [49]	Scott 2007 [35]	Richert 2011 [8]	Bauer 2011 [22]	Sullivan 2011 [21]	Erlandson 2012a [10]	Erlandson 2012b [18]	Cohen 2012 [45]	Beans 2013 [43]	Mbada 2013 [44]	Richert 2014 [9]	Erlandson 2014 [12]
1	Research question/objective clearly stated?	N	Y	Y	Y	Y	Y	Y	Y	N	Y	Y	Y	Y	Y	Y	Y	Y
2	Study population clearly specified and defined?	N	N	N	Y	N	Y	Y	Y	Y	Y	Y	Y	Y	Y	Y	Y	Y
3	Participation rate of eligible persons at least 50%?	CD	CD	CD	CD	CD	CD	CD	N	CD	CD	Y	Y	Y	Y	CD	N	CD
4	All subjects recruited from similar populations? Eligibility criteria pre-specified and applied uniformly?	NR	N	N	Y	NR	N	Y	Y	Y	Y	Y	Y	Y	Y	Y	Y	Y
5	Justification of sample size?	N	N	N	Y	N	N	N	N	N	N	N	N	N	N	N	N	N
6	Exposure(s) measured prior to outcome(s)?^a^	N	N	N	N	N	N	N	N	N	N	N	N	N	N	N	Y	N
7	Sufficient timeframe to see an association between exposure and outcome?^a^	N	N	N	N	N	N	N	N	N	N	N	N	N	N	N	Y	N
8	Different levels of the exposure measured, as related to the outcome?	Y	Y	Y	Y	Y	Y	Y	Y	Y	Y	Y	Y	Y	Y	Y	Y	Y
9	Exposure measures clearly defined, valid, reliable, and implemented consistently?	NR	Y	Y	Y	NR	Y	Y	Y	Y	Y	Y	Y	Y	NR	Y	Y	Y
10	Exposure(s) assessed more than once over time?^a^	N	N	N	N	N	N	N	N	N	N	N	N	N	N	N	CD	N
11	Outcome measures clearly defined, valid, reliable, and implemented consistently?	CD	NR	NR	NR	CD	Y	Y	NR	Y	NR	N	N	N	Y	Y	NR	CD
12	Outcome assessors blinded to exposure status?	NR	NR	NR	NR	NR	NR	NR	NR	NR	NR	NR	NR	NR	NR	NR	NR	NR
13	Loss to follow-up after baseline ≤20%?^a^	N	N	N	N	N	N	N	N	N	N	N	N	N	N	N	N	N
14	Key potential confounders measured and statistically adjusted for?	N	N	N	Y	N	Y	Y	Y	Y	Y	Y	Y	Y	Y	Y	Y	Y
	Total CAT score /14	1	3	3	7	2	6	7	6	6	6	7	7	7	7	7	8	6
	Total CAT %	7.14	21.43	21.43	50	14.29	42.86	50	42.86	42.86	42.86	50	50	50	50	50	57.14	42.86

*Abbreviations: CD* cannot determine, *NR* not reported

^a^Cross-sectional analyses provide weaker evidence than cohort studies regarding a potential causal relationship between exposures and outcomes. For cross-sectional analyses, the answer to Questions 6, 7, 10 & 13 should be “No”. All studies were cross-sectional, except for Richert 2014 (prospective longitudinal cohort)

#### Study sample description

Participant numbers varied from 19 to 447. Six studies did not include a control group [8–10, 12, 18, 35]. Mean ages ranged from 28 to 54.7 years. Two studies included males only [35, 43]. Only one study [44] was conducted in Sub-Saharan Africa. Table 2 summarizes the sample characteristics of all participants, while HIV-specific sample characteristics are presented in Table 3.

**Table 2:** Sample characteristics, all participants

Study ID	Country, setting	Serostatus & sample size (N)	Gender (%)	Age (years) (SD)	BMI (kg/m^2^) (SD)	Edu-cation (years) (SD)	Recreational drug use/Alcohol consumption/smoking (N)	Depression/PN/Other co-morbidities
Trenkwalder 1992 [46]	Germany, NR	HIV+ 50	M 96 F 4	42.5 (9.3)	NR	NR	4 / NR / NR	NR/Yes/Various neurological deficits
		HIV- 50	NR	37.5 (11.0)	NR	NR	NR / NR / NR	NR / NR / Healthy
Arendt 1994 [47]	Germany, NR	HIV+ 46	M 74 F 26	ASX: 36.33 (9.18) SX: 38.8 (8.38)	NR	NR	0 / 0 / NR	NR / No / HIV type-1-related encephalopathy (*n* = 10)
		HIV- 38	M 53 F 47	37.7 (10.21)	NR	NR	NR / NR / NR	NR / NR / Healthy
Beckley 1998 [50]	USA, NR	HIV+ 9	M 89 F 11	38.9 (10.7)	NR	NR	0 / 0 / NR	NR / No / PGL (*n* = 2); Opportunistic infection (*n* = 3)
		HIV- 10^a^	M 50 F 50	34.3 (7.8)	NR	NR	NR / NR / NR	NR / No / Healthy
Bauer 2005 [7]	USA, outpatient infectious disease clinics	HIV+ 90	M 39 F 61	NRx: 40 (7.2) NNRTI: 40 (5.9) PI: 39 (6.4)	NR	NRx: 11.5 (1.6); NNRTI: 11.6 (1.7); PI: 12.1 (2.6)	Large % Hx of drug abuse/NRx: 39.3%; NNRTI: 40%; PI: 35.1%/NR	NRx: 42.9%, NNRTI: 28%, PI: 35.1%/NR/Exclusion criteria eliminated major psychiatric-, medical- & neurological disorders
		HIV- 78	M 47.4 F 52.6	38 (7.1)	NR	12.6 (2.2)	Large % Hx of drug abuse/16.7%/NR	17.9% / NR / Healthy
Simmonds 2005 [49]	USA, out- patient AIDS facility	HIV+ 100	M 78 F 22	40.70 (7.49)	NR	NR	NR / NR / NR	NR / No / Exclusion criteria eliminated major medical & neurological disorders
		HIV- 105^a^	M 37 F 63	44.9 (14.7)	NR	NR	NR / NR / NR	NR / No / Healthy
Dellepiane 2005 [48]	Italy, NR	HIV+ 30	M 40 F 60	ASX: 28 (NR) AIDS: 32.8 (NR)	NR	NR	NR / NR / NR	NR / NR / Alcoholic cirrhosis (*n* = 1); no neurological or oto-neurological symptoms
		HIV- 55	M 64 F 36	35 (NR)	NR	NR	NR / NR / NR	NR / NR / Healthy
Scott 2005 [35]	USA, HIV clinic	HIV+ 27	M 100	48.7 (6.5)	24.2 (4.1)	NR	NR / NR / NR	NR / NR / Exclusion criteria eliminated major medical & neurological disorders
Richert 2011 [8]	France, HIV clinics	HIV+ 324	M 80 F 20	^b^47.6 (41.8, 53.9)	^b^22.5 (20.6, 24.6)	NR	NR / NR / NR	NR / 14% / Hepatitis B: 7%, Hepatitis C: 19%
Bauer 2011 [22]	USA, outpatient infectious disease clinics	HIV+ 121	M 58 F 42	BMI < 21: 39.4 (1.0); BMI 21–29: 40.9 (0.8); BMI > 29: 37.6 (1.2)	<21(*n* = 35); 21–29 (*n* = 61); >29(*n* = 25)	NR	No differences between groups/No differences between groups/NR	Significant differences (*P* < 0.05)/NR/Exclusion criteria eliminated major psychiatric-, medical- & neurological disorders
		HIV- 86	M 49 F 51	BMI < 21: 38.5 (1.3); BMI 21–29: 38.0 (1.1); BMI > 29: 36.6 (1.0)	<21(*n* = 2); 21–29 (*n* = 30); >29 (*n* = 35)	NR	No differences between groups/No differences between groups/NR	Significant differences (*P* < 0.05)/NR/Healthy
Sullivan 2011 [21]	USA, HIV clinics, local community	HIV+ 40	M 70 F 30	41 (NR)	M 25.4 (3.34); F 26 (3.16)	M 14.1 (3.05); F 13.8 (2.67)	NR / No differences between groups/M 43%, F 20%	BDI: M 10.5 (8.33); F 12.8 (9.26)/M 26%, F 17%/NR
		HIV- 83	M 48 F 52	44 (NR)	M 26.9 (4.83); F 24.7 (4.49)	M 15.9 (2.27); F 15.3 (2.00)	NR / No differences between groups/M 10%, F 0%	BDI: M 2.08 (2.33), F 2.9 (3.08)/NR/Without medical or psychiatric conditions
Erlandson 2012a [10]	USA, Infectious Diseases Group Practice clinic	HIV+ 359	M 85 F 15	^b^50.8 (47.7, 55.7)	NR	NR	IDU (<1%), Cocaine (<1%), Marijuana (23%) / >7 drinks/wk. (4%) / Current: 34%	NR /NR/NR
Erlandson 2012b [18]	USA, Infectious Diseases Group Practice clinic	HIV+ 359	M 85 F 15	52 (0.3)	NR	NR	Current IDU (<1%) / >7 drinks/wk.: Non-fallers (4%), Single fallers (7%), Re-fallers (2%) / Non-fallers (30%), Single-fallers (42%), Re-fallers (47%)	NR/NR/30% reported ≥1 falls during the past year (of those, 61% were recurrent fallers)
Cohen 2012 [45]	USA, multiple clinical subsites	HIV+ 247	M 51 F 49	48.9 (8.9)	NR	NR	NR/No/NR	NR/NR/Exclusion criteria eliminated spinal injury, vestibular impairment, use of narcotics, antihistamines or sedatives within 48 h of testing
		HIV- 200	M 84 F 16	54.2 (11.2)	NR	NR	NR/No/NR	NR/NR/NR
Beans 2013 [43]	USA, Baltimore VA Medical Center	HIV+ 45	M 100	54.4 (6.3)	<25 (51.1%) ≥25 (48.9%)	NR	NR/NR/69.0%	NR/NR/Diabetes 26.7%, Hepatitis C 71.1%, Hypertension 68.9%, Chronic Pulmonary Disease 20%, Dyslipidemia 36.4%, Anemia 24.4%
		HIV- 27	M 100	54.7 (6.2)	<25 (32.4%) ≥25 (67.6%)	NR	NR/NR/56.8%	NR/NR/Diabetes 18.9%, Hepatitis C 55.6%, Hypertension 73%, Chronic Pulmonary Disease 29.7%, Dyslipidemia 25.8%, Anemia 37.8%
Mbada 2013 [44]	Nigeria, Virology Research Clinic	HIV+ 37	M 40.5 F 59.5	35.68 (7.71)	22.77 (4.17)	NR	NR/NR/NR	NR/NR/NR
		HIV- 37	M 40.5 F 59.5	35.73 (7.88)	24.31 (4.24)	NR	NR/NR/NR	NR/NR/Healthy
Richert 2014 [9]	France, HIV clinics	HIV+ 178	M 81 F 19	^b^48 (43, 56)	^b^22.2 (20.5, 24.5)	NR	Prior IDU (14%)/NR/NR	NR/NR/Cerebral CDC stage C condition: 3%, Hepatitis B: 7%, Hepatitis C: 20%
Erlandson 2014 [12]	USA, Infectious Diseases clinic	HIV+ 359	M 85 F 15	52 (5.2)	26.4 (6.0)	NR	Current IDU (<1%) /NR/NR	NR/NR/NR

*Abbreviations: AIDS* Acquired Human Immunodeficiency Syndrome, *ART* antiretroviral therapy, *ASX* asymptomatic, *BDI* Beck Depression Inventory, *BMI* Body Mass Index, *CDC* Centre for Disease Control, *DAST-10* Drug Abuse Screening Test, *F* female, *HAART* highly active antiretroviral therapy, *HIV* human immunodeficiency virus, *Hx* history, *IDU* intravenous drug use, *M* male, *MAST* Michigan Alcoholism Screening Test, *MDD* Major Depressive Disorder, *N* number of participants, *NA* not applicable, *NNRTI* non-nucleoside reverse transcriptase inhibitor, *NR* not reported, *NRTI* nucleoside reverse transcriptase inhibitor, *NRx* no treatment, *PGL* Persistent generalized lymphadenopathy, *PI* protease inhibitor, *PN* peripheral neuropathy, *SD* Standard Deviation, *SX* symptomatic, *USA* United States of America, *WR* Walter Reed stages

^a^Retrospective control group of healthy volunteers from previous study

^b^Median (IQR)

**Table 3:** Sample characteristics, PLHIV

Study ID	Disease staging	CD4 cell count, cells/mm^3^ (SD)	Viral load (SD)	Treatment
Trenkwalder 1992 [46]	WR I-II (*N* = 17); WR III-V (*N* = 19); WR VI (*N* = 14)	NR	NR	NR
Arendt 1994 [47]	CDC II (*N* = 12); CDC III (*N* = 12); CDC IV C1 (*N* = 5); CDC IV C2 (*N* = 5); CDC IV D (*N* = 2); CDC IV B (*N* = 10)	NR	NR	NR
Beckley 1998 [50]	ASX (*N* = 2); CDC Stage A (*N* = 2); CDC Stage B (*N* = 2); CDC Stage C (*N* = 3)	Range 65–701; 5 participants had AIDS-defining CD4 counts (<200)	NR	Most were on zidovudine maintenance therapy
Bauer 2005 [7]	NR	NRx: 351 (282); NNRTI: 457 (375); PI: 320 (200)	*HIV burden × 1000 copies/ml:* NRx: 93.8 (163); NNRTI: 35.5 (102); PI: 20.1 (48.2)	NRx: *N* = 28; NNRTI: *N* = 25; PI: *N* = 37
Simmonds 2005 [49]	*Based on CD4 count* ASX (CD4 > 200) (*N* = 52); AIDS (CD4 < 200) (*N* = 48)	Range 189.83 (183.27) - 386.36 (302.39)	*Virions:* ASX 33545.25; AIDS 193401.00	NR
Dellepiane 2005 [48]	*CDC classification*ASX (*N* = 15)*;* AIDS (group IV) (*N* = 15)	NR	NR	NR
Scott 2007 [35]	NR	408 (293)	*log copies/ml* 2.18 (0.94)	All were on a NRTI-based regimen, with 82% receiving a PI as a third agent
Richert 2011 [8]	CDC category C: 23%	^a^520 (348, 709)	<500 copies/ml: 83%	89%
Bauer 2011 [22]	NR	BMI <21: 280 (52); BMI 21–29: 422 (40); BMI > 29: 361 (64)	*Log10 viral load* BMI <21: 3.06 (0.34); BMI 21–29: 2.19 (0.26); BMI > 29: 2.08 (0.39)	*% no ART/NNRTI-based ART/ PI-based ART:* BMI <21: 38.2/26.5/35.3; BMI 21–29: 31.7/26.7/ 41.7; BMI > 29: 37.0/18.5/44.4
Sullivan 2011 [21]	NR	M 537.4 (258.97); F 583.4 (103.55)	M 13597.6 (4654.88); F 4609.7 (3226.36)	HAART: *N* = 25; Non-HAART: *N* = 6; NRx: *N* = 9
Erlandson 2012a [10]	NR	^a^551 (361, 768)	Detectable (≥48 copies/mL): 5%	NR
Erlandson 2012b [18]	NR	594 (16)	95% had plasma HIV-1 RNA < limits of detection	Any didanosine: Non-fallers: 57 (23); Single fallers: 10 (23); Recurrent fallers: 24 (36) Any stavudine: Non-fallers: 93 (37); Single fallers: 22 (51); Recurrent fallers: 33 (50) Efavirenz: Non-fallers: 86 (34); Single fallers: 10 (23); Recurrent fallers: 22 (33)
Cohen 2012 [45]	NR	556.4 (284)	*Log*_*10*_ *HIV RNA:* ^a^3.50 (2.68, 4.42)	HAART: 76.9%
Beans 2013 [43]	NR	^a^445 (265, 531)	Non-detectable (<400 copies/ml): 91%	Majority were receiving cART
Mbada 2013 [44]	All: Clinical stage I of HIV/AIDS (ASX HIV infection, with PGL)	NR	NR	100% HAART
Richert 2014 [9]	CDC stage C 24%	^a^506 (340, 715)	HIV RNA level < 500 copies/ml: 84%	89% on ART
Erlandson 2014 [12]	NR	594 (303)	HIV-1 RNA < limits of detection: 95%	All participants taking effective cART

*Abbreviations: AIDS* acquired immunodeficiency syndrome, *ART* antiretroviral therapy*, ASX* asymptomatic*, BMI* Body Mass Index*, cART* combination antiretroviral therapy, *CDC* Centre for Disease Control*, F* female, *HAART* highly active antiretroviral therapy*, HIV* human immunodeficiency virus, *IQR* interquartile range, *M* male*, N* number of participants, *NA* not applicable, *NNRTI* non-nucleoside reverse transcriptase inhibitor*, NR* not reported*, NRTI* nucleoside reverse transcriptase inhibitors, *NRx* no treatment*, PGL* Persistent generalized lymphadenopathy, *PI* protease inhibitor*, PLHIV* people living with HI, *SD* standard deviation, *SX* symptomatic, *WR* Walter Reed staging

^a^Median (IQR)

#### Study design, aims and outcomes

Sixteen studies were cross-sectional, and one was a prospective cohort [9]. Study aims varied (Table 4), but all included objective measurement of balance and/or gait as part of the primary aim. Balance was assessed using both clinical and instrumented tests. All studies used timed clinical tests for assessing gait. No studies assessed gait kinetics or kinematics. Outcomes varied substantially. Table 5 (balance) and Table 6 (gait) present the outcomes assessed per study. Summaries of the results for individual outcomes are presented briefly in Table 7 (balance) and Table 8 (gait), and presented in more detail as additional files (see Additional file 1 for balance and Additional file 2 for gait).

**Table 4:** Study aims

Study ID	Design	Aim
Trenkwalder 1992 [46]	Cross-sectional	To measure postural performance quantitavely in PLHIV (in different disease stages) versus seronegative controls, using a force plate.
Arendt 1994 [47]	Cross-sectional	To determine if stance control is impaired in early versus late HIV infection, using a force plate, and to compare results with the COG patterns in pyramidal or extrapyramidal disease.
Beckley 1998 [50]	Cross-sectional	To evaluate postural reflexes with EMG in PLHIV without obvious neurological disease, in order to determine whether postural reflexes are early markers of CNS involvement.
Bauer 2005 [7]	Cross-sectional	To assess sensorimotor function in PLHIV and seronegative controls.
Simmonds 2005 [49]	Cross-sectional	To characterize physical performance in PLHIV, and to examine group differences by pain and fatigue.
Dellepiane 2005 [48]	Cross-sectional	To investigate whether posturography can detect the presence of possible disorders of the vestibulo-spinal reflex.
Scott 2007 [35]	Cross-sectional	To determine the extent of neuromuscular activation of selected lower limb muscles of male PLHIV receiving ART, and its relationship to performance in clinical functional tests.
Richert 2011 [8]	Cross-sectional	To provide standardized assessments of locomotor function in PLHIV, focusing on lower limb muscle performance and balance, and on potential determinants of functional impairment.
Bauer 2011 [22]	Cross-sectional	To compare balance and gait in participants who differ in BMI and the presence or absence of HIV/AIDS.
Sullivan 2011 [21]	Cross-sectional	To investigate whether infratentorial brain volume would be marked by regional tissue shrinkage in PLHIV versus seronegative controls, and whether tissue deficits would be related to impairment in postural stability or psychomotor speed, using structural MRI and quantitative tests of postural stability, finger movement, psychomotor speed and dexterity.
Erlandson 2012a [10]	Cross-sectional	To compare the FFP, SPPB, and 400-m walk in PLHIV.
Erlandson 2012b [18]	Cross-sectional	To determine fall-rate and -risk factors among PLHIV by correlating fall history, medical diagnoses, and functional tests.
Cohen 2012 [45]	Cross-sectional	To determine whether PLHIV on HAART had an increased prevalence of vestibular disorders versus seronegative controls, using standard screening tests of vestibular and balance function.
Beans 2013 [43]	Cross-sectional	To compare locomotor function in male PLHIV versus seronegative controls, and test the association with aerobic exercise capacity.
Mbada 2013 [44]	Cross-sectional	To compare HRQOL and a performance-based measure of functional capacity between a homogenous sample of clinical stage I PLHIV versus seronegative controls.
Richert 2014 [9]	Prospective cohort	To prospectively assess the changes in locomotor function in PLHIV over time and to evaluate the determinants of variations in lower limb muscle performance.
Erlandson 2014 [12]	Cross-sectional	To assess the impact of physical function impairments on HRQOL in PLHIV using ART.

*Abbreviations*: *ART* antiretroviral therapy, *BMI* body mass index, *CNS* central nervous system, *COG* centre of gravity, *EMG* electromyography, *FFP* Fried’s Frailty Phenotype, *HRQOL* health-related quality of life, *PLHIV* people living with HIV, *SPPB* Short Physical Performance Battery

**Table 5:** Studies assessing balance outcomes

		Trenkwalder 1992 [46]	Arendt 1994 [47]	Beckley 1998 [50]	Bauer 2005 [7]	Dellepiane 2005 [48]	Simmonds 2005 [49]	Richert 2011 [8]	Bauer 2011 [22]	Sullivan 2011 [21]	Cohen 2012 [45]	Erlandson 2012a [10]	Erlandson 2012b [18]	Richert 2014 [9]	Erlandson 2014 [12]	Total studies assessing outcome
Balance outcome	Mean sway path (m/min)	X														1
	Sway velocity (m/s)		X													1
	Sway area (μVxs)		X			X										2^a^
	AP					X										1
	LAT					X										1
	AP/LAT quotient		X			X										2^b^
	Romberg ratio of sway velocity; RW		X			X										2^a^
	Romberg area of sway; RA					X										1
	Way					X										1
	SOT sway strategy score								X							1
	SOT EQ				X											1
	SOT number of falls; time before fall				X											1
	FBOS; LOS				X				X							2^c^
	Latencies of postural reflexes (ms)		X	X		X										3^a^
	Duration of postural reflexes					X										1
	Amplitude of postural reflexes					X										1
	Area of single EMG potential					X										1
	Normalized amplitude of ML-response			X												1
	Standardized LL Z-scores			X												1
	Romberg ECF (sec)										X					1
	Tandem stance (sec)									X						1
	Single leg stance time (sec)							X	X	X				X		4^d^
	Berg balance score							X								1
	TUG time (sec)							X						X		2^e^
	5STS time (sec)							X	X					X		3^f^
	5STS pace (rises/s)														X	1
	360° turn time								X							1
	Walk heel-to-toe (number of steps)									X						1
	Forward reach distance (cm)						X	X								2^g^

*Abbreviations: 5STS* 5-times sit-to-stand test, *AP* average velocity in an anterior-posterior direction, *cm* centimeters, *ECF* eyes-closed-on-foam, *EMG* electromyography, EQ equilibrium quotient, *FBOS* functional base of support, *LAT* average velocity in a medial-lateral direction, *LL* long loop, *LOS* limits of stability, *m* meters, *min* minute, *ML* medium loop, *ms* millisecond, *RA* Romberg area of sway, *RW* Romberg ratio of sway velocity, *sec* second, *SOT* sensory organization test, *TUG* timed-up-and-go test

^a^Meta-analysis performed

^b^Meta-analysis not done as Arendt 1994 does not report SD values

^c^Meta-analysis not done as Bauer 2005 does not report values for control group

^d^Meta-analysis not done due to heterogeneity in methodologies: Richert 2011 uses established normative values as comparison; Sullivan 2011 uses max time of 60 s, Richert 2014 has no comparison values

^e^Meta-analysis not done as Richert 2014 has no comparison group

^f^Meta-analysis not done as Richert 2011 & 2014 has no comparison groups

^g^Meta-analysis not done as Richert 2011 uses established normative values as comparison

**Table 6:** Studies assessing gait outcomes

		Bauer 2005 [7]	Simmonds 2005 [49]	Scott 2007 [35]	Richert 2011 [8]	Bauer 2011 [22]	Erlandson 2012a [10]	Erlandson 2012b [18]	Beans 2013 [43]	Mbada 2013 [44]	Richert 2014 [9]	Erlandson 2014 [12]	Total studies assessing outcome
Gait outcomes	Gait speed (m/s), preferred and/or fast					X	X	X			X	X	5^a^
	Timed gait (sec)	X	X						X				3^b^
	Cadence (time in sec for 5 steps), fast and preferred	X				X							2
	Gait initiation time (sec), fast and preferred					X							1
	6MWD		X	X	X				X	X	X		6^c^

*Abbreviations: 6MWD* 6-min walk distance, *m* meter*, sec* second

^a^Meta-analysis not possible as Bauer 2005 did not report mean results for gait speed and Richert 2014 included no comparison group or norm values

^b^Meta-analysis not possible as Bauer 2005 did not report mean for gait speed and Erlandson 2012a, 2012b & 2014 included no comparison groups or norm values

^c^Only 2 out of 6 studies included in meta-analysis, as Beans 2013 included men only, Scott 2007 & Richert 2011 & 2014 included no comparison groups or norm values (note heterogeneity between samples of the 2 studies included meta-analysis)

^d^Meta-analysis not possible, as Bauer 2005 did not report mean results for cadence or gait initiation time

**Table 7:** Summary of objective balance outcomes and results

Study ID	Results	Method of measurement	Outcomes assessed
Trenkwalder 1992 [46]	^b^,^a^	4 conditions on force plate: Bilat stance EO + stable; Bilat stance EC + stable; Bilat stance EO + foam; Bilat stance EC + foam.	Mean sway path (m/min): EO & EC + foam^b^(all PLHIV except WR I-II)/EC + stable or foam^b^(all PLHIV)/All other conditions ^a^
Arendt 1994 [47]	^b^,^a^	2 conditions on force plate: Bilateral stance EO; Bilateral stance EC.	Sway velocity (m/s)^b^ / AP/LAT quotient ^a^
Beckley 1998 [50]	^b^,^a^	Leg reflexes elicited in participants while standing upright on movable force plate - surface EMG recordings obtained from left tibialis anterior and medial gastrocnemius	Onset latencies (SL, ML and LL) (ms) / Normalized amplitude of ML^a^/LL-amplitude scaling (predictable ^a^; unpredictable ^b^)
Bauer 2005 [7]	^b^,^a^	1) SOT, 3 conditions: EO, EC, inaccurate visual input 2) Forward/backward lean tests 3) (Single-leg stance test)	1) SOT, for each condition: EQ. (EO^a^, EC^b^, inaccurate^a^)/Number of falls^a^/Time before a fall (seconds)^a^ 2) FBOS (Lean amplitude/ft length)^b^ 3) (Single Leg Stance time (s) - results not presented)
Simmonds 2005 [49]	^a^	Loaded forward reach Unloaded forward reach	Distance reached (cm)^a^
Richert 2011 [8]	^a^,^c^	1) BBS 2) TUG test 3) FR test 4) SLS, EC 5) 5STS	1) Berg score^a^ 2) TUG time (sec)^a^ 3) Reach distance (cm)^a^ 4) SLS time (sec)^c^ 5) 5STS time (sec)^c^
Dellepiane 2005 [48]	^b^, ^a^	1) Static posturography: Romberg’s position on force plate; EO & EC 2) Dynamic posturography: EO & EC; leg reflexes elicited via sudden tilts of moveable force plate, EMG recorded	1) Static: Way (EO & EC, SX^b^), Area, AP (ASX in EC^b^, SX in EO^b^ & EC^b^), LAT (SX in EC^b^), AP/LAT^a^, RW, RA^b^ 2) Dynamic (SL, ML and LL): Latency (SL: EO & EC, all HIV groups^b^) (ML: EO, SX, both legs^b^; EO, ASX, left leg ^b^; EC, all groups^a^) (LL: EC, SX^b^; EC, ASX^a^)/Duration (SL: EO, all PLHIV ^a^; EC, SX, left leg^b^) (ML: EO, all PLHIV^a^; EC, all PLHIV, bilat^b^) (LL, EC, all PLHIV^b^) /Amplitude^a^/Area of single EMG^a^
Bauer 2011 [22]	^b^, ^a^	1) SOT, 3 conditions: EO, EC, inaccurate visual input 2) Forward/backward lean tests 3) SLS test 4) 360-degree turn test 5) 5STS test	1) SOT: Dependent variables calculated for each condition were: EQ (EC^b^, inaccurate input^b^) Sway strategy score (EC^b^) 2) LOS (lean amplitude/ft length)^b^ 3) SLST time (seconds) (only obese PLHIV, non-preferred leg^b^) 4) 360 deg. turn time (seconds) (only obese PLHIV^b^) 5) 5STS time (seconds)^a^
Sullivan 2011 [21]	^b^, ^a^	Walk-a-Line Battery. Conditions: Stand Heel-to-Toe; Walk Heel-to-Toe; and SLS.	1) Stand Heel-to-Toe time (seconds)^a^ 2) SLS time (seconds) (non-preferred leg^b^) 3) Walk-Heel-to-Toe - number of steps out of 10 (EC^b^)
Cohen 2012 [45]	^c^	Romberg tests on stable and on foam, 4 conditions: EO + stable, EC + stable, EO + foam, EC + foam.	Romberg time, EC + foam (seconds)^c^
Erlandson 2012a [10]	^c^	Tandem stand and 5STS as part of SPPB	5STS time (part of SPPB score)^c^/Tandem stance time (part of SPPB score)^c^
Erlandson 2012b [18]	^c^	Tandem stand and 5STS as part of SPPB	5STS time (part of SPPB score)^c^/Tandem stance time (part of SPPB score)^c^
Richert 2014 [9]	^c^	1) 5STS test 2) TUG test 3) SLS test	1) 5STS time (seconds)^c^ 2) TUG time (seconds)^c^ 3) SLS time (seconds)^c^
Erlandson 2014 [12]	^c^	5STS	5STS pace (rises/s)^c^

Outcomes included in meta-analyses are not included in this table

*Abbreviations: 5STS* 5-times-sit-to-stand*, AP* Average velocity in anterior-posterior direction*, ASX* asymptomatic; *BBS* Berg Balance Scale*, Bilat* bilateral*, COP* center of pressure*, deg.* degree*, EC* eyes closed*, EMG* electromyography, *EO* eyes open*, EQ* equilibrium quotient, *FBOS* functional base of support*, FR* functional reach*, LAT* average velocity in medial-lateral direction*, LL* long loop*, LOS* limits of stability*, ML* medium loop, *PLHIV* people living with HIV, *RW* Romberg index reported to way = ratio of way with EO & EC, *RA* Romberg index reported to area = ratio of area with EO & EC, *SL short loop, SLS* single leg stance*, SOT* sensory organization test*, SX* symptomatic, *TUG* timed-up-and-go

^a^no significant difference between PLHIV and controls

^b^PLHIV significantly impaired compared to controls or normative reference values

^c^No comparison provided/impairment quantified by reporting proportion of PLHIV with deficits

**Table 8:** Summary of objective gait outcomes and results

Study ID	Results	Method of assessment	Spatiotemporal outcome
Bauer 2005 [7]	^a^	8-m walk (preferred and fast)	Gait speed: time (sec) to cover distance^a^ Cadence (time in sec for 5 steps)^a^
Simmonds 2005 [49]	^b^	50-ft (15.24-m) walk (preferred and fast)	Gait speed: time (sec) to cover distance^b^
Scott 2007 [35]	^b^	6MWD	Distance covered (m) in 6 min^b^
Richert 2011 [8]	^c^	6MWD	Distance covered (m) in 6 min^c^
Bauer 2011 [22]	^b^	8-m walk (preferred and fast)	Preferred^b^ and fast gait initiation time (sec) Fast^b^ and preferred gait speed (m/s) Fast and preferred cadence (time in sec for 5 steps)
Erlandson 2012a [10]	^c^	4-m walk as part of SPPB 400-m walk (fast)	Only presented as part of SPPB score Gait speed (m/s)^c^
Erlandson 2012b [18]	^c^	1) 4-m walk as part of SPPB 2) 400-m walk (fast)	1) Only presented as part of SPPB score 2) Gait speed (m/s)^c^
Beans 2013 [43]	^d^,^a^	1) 6MWD 2) 400-m long distance corridor walk	1) Distance covered (m) in 6 min^a^ 2) Gait speed: time (sec) to cover distance^d^
Richert 2014 [9]	^b^	1) 6MWD 2) 10-m walk	1) Distance covered (m) in 6 min^b^ 2) Gait speed (m/s)
Erlandson 2014 [12]	^c^	400-m walk (fast pace)	Gait speed (m/s)^c^

Outcomes included in meta-analyses are not included in this table

*Abbreviations: 6MWD* 6 min walk distance*, m* meters*, min* minutes*, sec* seconds, *SPPB* short physical performance battery

^a^No significant difference between PLHIV and controls

^b^PLHIV significantly impaired compared to controls or normative reference values

^c^No comparison provided/impairment quantified by reporting proportion of PLHIV with deficits

^d^Controls performed worse

##### Static balance

Five studies assessed static balance using clinical tests. One study [45] assessed Romberg eyes-closed-on-foam and found the frequency of impairment to be higher in PLHIV. Tandem stance time was normal in PLHIV [21]. Four studies assessed single leg stance time [8, 9, 21, 22]. Impairments were noted either only with eyes closed, or with synergistic obesity, or when standing on the non-preferred leg (eyes open and closed).

One study [46] assessed COP sway path using a force plate, and found the incidence of increased values to be larger in advanced stages of infection and task difficulty. Sway velocity was examined by another study [47]. A significant increase was found in neurologically symptomatic PLHIV regardless of visual condition, and about 25% of PLHIV with asymptomatic HIV infection also demonstrated increased values.

Average velocity in anterior-posterior (AP) and average velocity in lateral (LAT) directions were assessed by one study [48]. PLHIV with asymptomatic HIV infection had significantly increased AP only in the eyes closed condition, while PLHIV with symptoms of chronic HIV disease had significantly increased AP both with eyes open and eyes closed, as well as significantly increased LAT (only with eyes closed).

Two studies [47, 48] assessed the coefficient of the preferential direction of movement (AP/LAT ratio) and found this to be normal in PLHIV. Romberg ratio of area (RA) as well as Way (average velocity of movement) was found to be increased in all HIV groups [48].

Sensory Organisation Test (SOT) sway strategy score was found to be lower (which is worse, as it indicates more reliance on the hip strategy as opposed to the ankle strategy) for bilateral stance (eyes closed) in PLHIV [22]. Two studies [7, 22] reported on SOT Equilibrium Quotient (EQ) and reported significant impairments in PLHIV during the most difficult SOT subtests (eyes closed or inaccurate visual input).

**Fig. 2:**
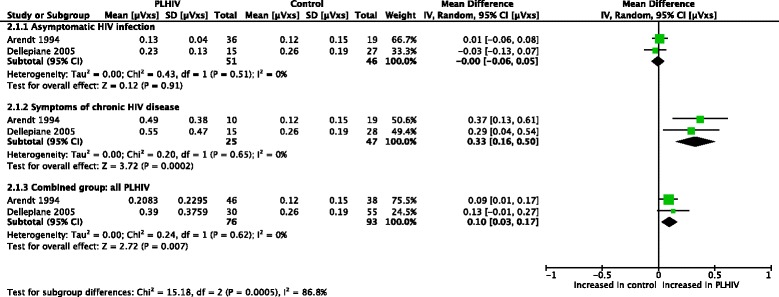
Meta-analysis of sway area (μVxs) in PLHIV, eyes open

**Fig. 3:**
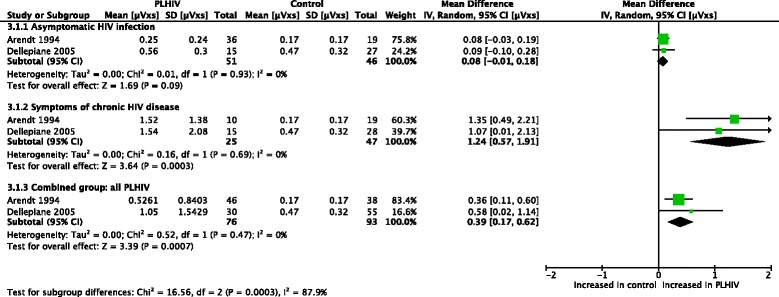
Meta-analysis of sway area (μVxs) in PLHIV, eyes closed

Meta-analyses (Figs. 2 and 3) were performed for postural sway area [47, 48]. With eyes open, asymptomatic PLHIV and controls had similar sway areas, while PLHIV with symptoms of chronic HIV disease demonstrated a significant increase. Overall, sway area was significantly increased in PLHIV (combined group of those with and without symptoms of HIV). With eyes closed, PLHIV with asymptomatic HIV infection had normal sway areas, while PLHIV with symptoms of chronic HIV disease demonstrated a significant increase. Overall, sway area was increased in PLHIV.

Thus, the observed overall treatment effect in the combined group differed across the different subgroups. Homogeneity seems to exist between the sample estimates within the subgroups (I^2^ = 0% for all groups), while a significant interaction existed between the subgroups (I^2^ = 86.4% & 87.9% for the two outcomes, respectively), suggesting that the population parameters estimated by the subgroups are different. It should however be noted that the conjecture about homogeneity between sample estimates in these subgroup does not necessarily mean that the presence/absence of symptoms in PLHIV fully explains the heterogeneity observed across studies. In fact, the small number of studies and sample sizes for these outcomes might not provide adequate statistical power in demonstrating heterogeneity.

**Fig. 4:**
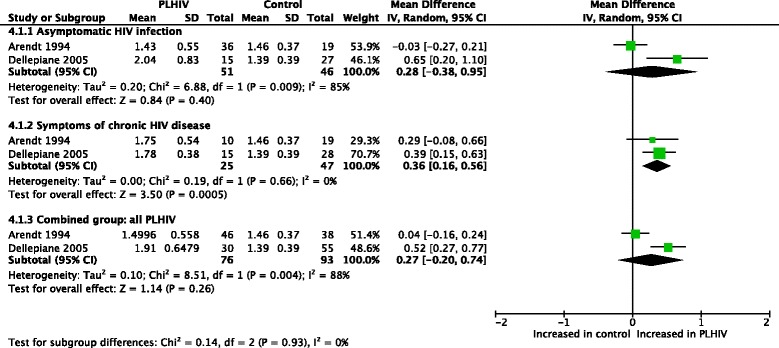
Meta-analysis of Romberg ratio of sway velocity in PLHIV

A meta-analysis (Fig. 4) was done for Romberg ratio of sway velocity (sway with eyes closed/sway with eyes open) [47, 48]. PLHIV with asymptomatic HIV infection had normal values, while PLHIV with symptoms of chronic HIV disease demonstrated a significantly larger Romberg ratio (which is worse as it indicates a higher amount of visual dependency). Overall, Romberg ratios were similar between the combined group of PLHIV and controls.

Substantial heterogeneity was found within the combined group (I^2^ = 88%, *p* = 0.004 and I^2^ = 91%, *p* = 0.00001, respectively). When splitting the subgroups according to presence of symptoms, PLHIV with asymptomatic HIV infection still showed evidence of high heterogeneity and non-significant results regarding impairment, while symptomatic PLHIV produced no evidence of heterogeneity (I^2^ = 0%) whilst showing significant impairment for this outcome.

The high heterogeneity that exists particularly in the asymptomatic subgroup of PLHIV might be attributed to differences in the study populations used by the two studies. Differences existed in the sample sizes used (36 asymptomatic PLHIV in Arendt (1994) [47] versus only 15 in Dellepiane et al. (2005) [48]). Also, the age of the asymptomatic participants in these studies differed (mean of 36.33 versus 28 years). Finally, although both studies had similar definitions of “symptomatic” participants, only Arendt (1994) further classified the asymptomatic group into CDC disease stages.

##### Dynamic balance

Both the Berg Balance Scale [8] and Timed-Up-And-Go (TUG) test [8, 9] were normal in PLHIV. For 5-Times-Sit-To-Stand (5STS) time, one study [22] found no group differences, while another [8] reported poor performance in PLHIV. The prospective cohort [9] reported an impaired 5STS time at baseline, and that 31% of PLHIV had a decline in performance over 1 year that was greater than the empirically defined threshold. Only PLHIV who were also obese performed worse in the 360-Degree-Turn test [22]. Walk-Heel-To-Toe was significantly impaired in PLHIV with eyes closed [21]. Two studies [8, 49] evaluated forward-reach distance, with no significant deficits noted.

The Functional Base of Support (FBOS) or Limits of Stability (LOS) tests were assessed by two studies [7, 22]; both reported significant impairments in all PLHIV.

Duration of postural reflexes was assessed by one study [48]. With eyes closed, there was a significant reduction for medium loop (ML) duration and long loop (LL) duration in all HIV groups. Amplitude of postural reflexes and area of single electromyography (EMG) potential were normal in PLHIV [48]. Neurologically intact PLHIV showed abnormal regulation of postural reflexes (LL amplitude scaling) under unpredictable, but not predictable, perturbations [50].

**Fig. 5:**
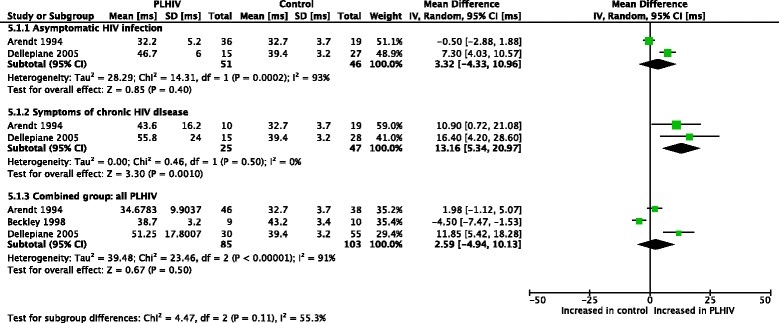
Meta-analysis of left leg postural reflex latencies in PLHIV: short loop latencies (ms)

**Fig. 6:**
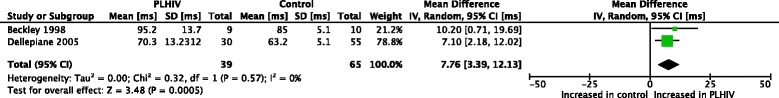
Meta-analysis of left leg postural reflex latencies in PLHIV: medium loop latencies (ms)

**Fig. 7:**
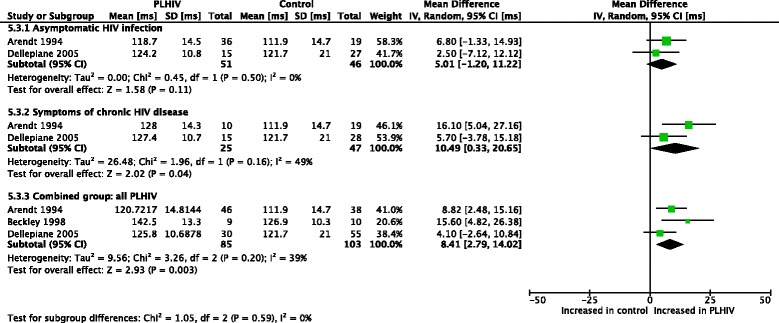
Meta-analysis of left leg postural reflex latencies in PLHIV: long loop latencies (ms)

**Fig. 8:**
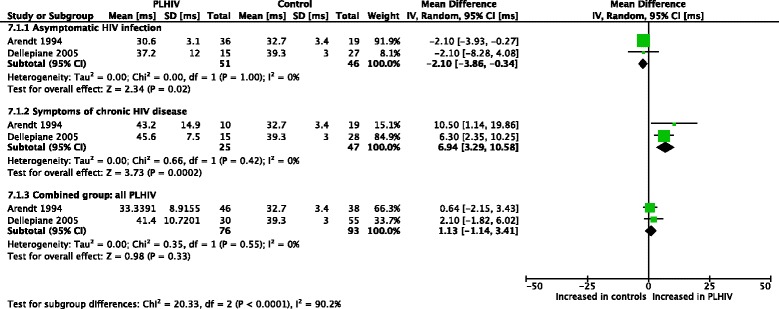
Meta-analysis of right leg postural reflex latencies in PLHIV: short loop latencies (ms)

**Fig. 9:**
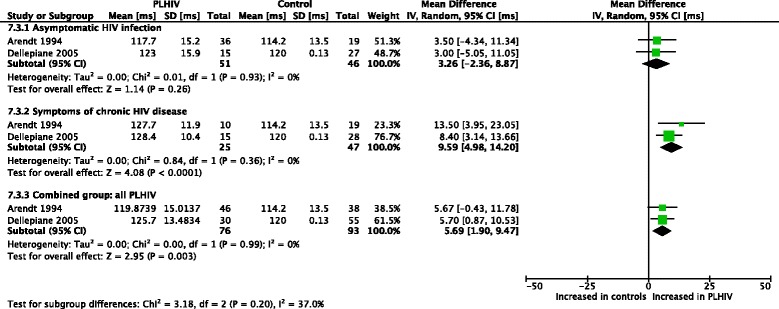
Meta-analysis of right leg postural reflex latencies in PLHIV: long loop latencies (ms)

Meta-analyses were conducted for postural sway latencies [47, 48, 50] (Figs. 5, 6, 7, 8 and 9). For the left leg, short loop (SL) latencies for combined PLHIV groups were normal, with significantly increased values only in PLHIV with symptoms of chronic HIV disease upon further analysis. These findings were similar for the right leg. ML latencies, only assessed in two of the studies [48, 50] and only for the left leg, were significantly increased in combined PLHIV groups. In both legs, LL latencies were significantly increased in symptomatic, but not asymptomatic, PLHIV. The combined PLHIV group still showed a significant increase in LL latencies.

##### Gait

Gait speed was assessed in eight studies [7, 9, 10, 12, 18, 22, 43, 49]. PLHIV demonstrated slowing of fast gait speeds [18, 43, 49]. One study [7] found no significant differences between PLHIV and controls, regardless of pace.

**Fig. 10:**
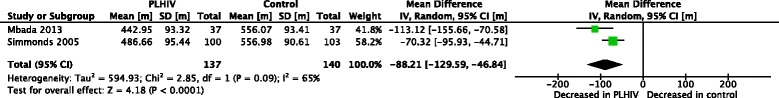
Meta-analysis of 6-Minute Walk Distance (m) in PLHIV

Meta-analysis [44, 49] (Fig. 10) indicated that 6-Minute Walk Distance (6MWD) was significantly shorter (worse) in PLHIV compared to controls. The likelihood of high heterogeneity in this meta-analysis should be considered (I^2^ = 65%, *p* = 0.09) and might be due to the use of historical controls in one study [49] and differences in disease staging between the two studies. Among the un-pooled studies, three reported a decreased (worse) 6MWD [8, 9, 35], and one study found no impairment in PLHIV [43]. One study reported no impairments in PLHIV in cadence [7] and another reported that only PLHIV who were also obese were significantly impaired [22]. This study also reported that PLHIV had significantly delayed (worse) normal gait initiation time.

##### Falls

One study [50] reported that fall incidence during unpredictable perturbations was similar in PLHIV versus controls. Similarly, another study [7] found no group differences in falls during SOT conditions. In contrast, one study reported a similar fall rate in middle-aged PLHIV (mean 52.0 years) and seronegative older adults (≥65 years) [18]. Impaired balance was a major associated factor. In addition, recurrent fallers had significantly slowed gait versus non-fallers. Furthermore, in a prospective cohort [9], it was reported that 12% of PLHIV experienced a minimum of one fall in the previous year. In PLHIV with recurrent falls, baseline 5STS time and 6MWD were significantly impaired, compared to non-fallers.

##### Measurement conditions and task difficulty

Twelve studies included some form of increasing task difficulty, such as different visual input, stable versus unstable support surfaces, decreased base of support, predictable and unpredictable external perturbations, and walking at preferred versus fast gait speeds. Of these, nine (75%) demonstrated that both balance and gait impairments were more evident in more difficult task conditions, when comparing PLHIV to controls [7, 10, 18, 21, 22, 45, 46, 49, 50].

##### Disease severity

Fifteen studies reported on the relationship between HIV-disease severity and locomotor performance. Of these, eight (53%) indicated a relationship between HIV-disease severity and impairments [7, 8, 10, 35, 46–48, 50]. In contrast, seven studies (46%) found no significant differences based on CD4 counts or viral loads [9, 21, 22, 43, 45, 49, 50].

##### Treatment effects

Seven studies reported on the relationship between ART and impairments in gait and/or balance, and none of these found any association [7–10, 21, 22, 35].

##### Peripheral neuropathy

Five studies reported on the association between peripheral neuropathy and impairments in gait and/or balance in PLHIV, and none of these found statistically significant correlations between peripheral neuropathy and impairments of gait, dynamic balance or static balance [7, 8, 21, 46, 47].

## Discussion

The aim of this review was to establish the current state of knowledge regarding objective impairments of gait and balance in PLHIV, and to emphasize those which could contribute to increased fall risk. To the authors’ knowledge, this is the first work to do so. Our findings indicate that certain aspects of gait and balance are impaired in middle-aged PLHIV, resembling those proven to predict increased fall risk in elderly populations.

The methodological quality of articles ranged from fair to low, partly as a direct consequence of observational design. Earlier studies in particular had a high risk of selection bias due to omitting important information such as participant demographics and exclusion criteria. The psychometric properties of the different tests used to assess outcomes have not yet been evaluated in PLHIV; therefore, they cannot be assumed to be valid and reliable in this specific population. Balance and gait in PLHIV may be influenced by various factors apart from HIV-status. Although studies on average controlled and adjusted for many key confounders such as age, gender, BMI, markers of HIV and co-morbidities, very few reported on covariates such as level of education or adherence to treatment, and none on level of physical activity.

Various gait and balance parameters, including slowed gait speed [38], cadence [51], slowed gait initiation time [52] slowing of postural reflexes [53], and increased COP displacement and velocity [54] have been established to be associated with increased risk of falls in the elderly. Similarly, some of these variables are associated with risk of falls in PLHIV, namely slowed gait speed and impaired dynamic balance [18]. It has been reported that the best fall risk predictors in PLHIV are those proven to be predictors of fall risk in the elderly [18].

### Static balance

Static balance is often quantified in terms of COP movement [55], which reflects neuromuscular control to keep the center of mass (COM) within the base of support’s limits of stability [56–58]. Increased COP movement and velocity is associated with increased fall risk in the elderly [54]. In this review, evidence of increased postural sway or velocity was found in all studies evaluating these parameters, especially under challenging conditions [46–48], and was confirmed by meta-analyses. Impaired COP sway in PLHIV with asymptomatic HIV infection may suggest early involvement of postural control due to direct infection of the CNS by HIV. However, in neurologically symptomatic PLHIV, it cannot be assumed that anatomical structures or direct HIV-involvement of the CNS causes the observed deficits [46]. Lower limb muscle impairment might impair a person’s ability to correct a shift in the body’s COP to effectively prevent a fall [59]. In the elderly, it has been proposed that increased COP movement may be interpreted as an increase in hip abductor muscle activity to control postural stability on the medial-lateral direction [59]. It has also been suggested that decreased postural control with larger body sway increases tibialis anterior/soleus muscle co-activation, inducing the hip-strategy to preserve balance [60]. Greater co-activation may be partly be a compensation for decreased lower limb muscle strength and power [61]. As lower limb muscle impairments occur in PLHIV, this might contribute to the impaired COP parameters observed. HIV-associated vestibular dysfunction has also been reported [62]. Vestibular disorders have a deleterious effect on postural stability [63]. However, vestibular conditions are not characterised by impaired COP excursion, but rather by an increased frequency of movement, indicating poor control of COP [63].

A lower sway strategy score (the relative amount of high-frequency ankle versus low-frequency hip movement) for bilateral stance with eyes closed was found in PLHIV, albeit only reported in a single study in this review [22], indicating a heavier reliance on the hip-strategy. In the general population, intact individuals will change their balance strategy from the normally employed ankle strategy, to relying on the hip strategy [56] when faced with more challenging conditions. Individuals with impaired balance, who already rely more heavily on the hip strategy, are less able to adapt to challenging conditions [56].

The SOT Equilibrium Quotient (EQ) is a calculation of the average COP sway, with lower EQ scores having been associated with increased fall risk in the elderly [64]. The two studies evaluating this outcome [7, 22] reported significantly lower EQ scores in PLHIV, especially with more challenging conditions.

Reduced single leg stance time is predictive of some (i.e. injurious), but not all, falls in the elderly [65]; however, the clinical value of this test might be called into question. The test might suffer from learning effects [66], leading to possible ceiling effects even in individuals with substantial impairment when only performed as a clinical test. Due to differences in the reporting of results among the included studies assessing this outcome, it is difficult to draw conclusions regarding impairment and the value of the test in PLHIV.

### Dynamic balance

Dynamic balance is often assessed using dynamic posturography, which involves external perturbations being induced while a person tries to maintain an upright posture [56]. A common postural synergy in this scenario is the distal-to-proximal ankle strategy, involving a short loop (SL) and medium loop (ML) response in the gastrocnemius, followed by a long loop (LL) response in the tibialis anterior [67, 68]. Prolonged stance-stabilizing LL responses have been documented in elderly fallers [69]. Meta-analyses indicated that LL latencies were increased in symptomatic but not asymptomatic PLHIV, and upon combining all groups of PLHIV, LL latencies were still significantly increased. It is suggested that the early-observed prolonged LL latencies in PLHIV with asymptomatic HIV infection may indicate alterations in the central dopaminergic system (basal ganglia, caudatus nucleus and nigrostriatal system) [48].

Scaling of LL latency-amplitude, referring to the ability to adjust the size of posturally stabilizing reflexes and another important factor associated with falls [53], was assessed in one study [50]. Neurologically intact PLHIV showed abnormal postural reflex regulation under unpredictable, but not predictable, perturbations. Under random conditions, PLHIV automatically selected a LL response of a relatively similar size to one needed for medium perturbations. This response may not be sufficient to correct for large perturbations, leading to an increased risk of falling. However, the authors noted that the impairment in PLHIV was “mild” and did not appear clinically significant in early HIV infection.

The Limits of Stability (LOS) or Functional Base of Support (FBOS) test involves instrumented measurement of a forward leaning task and evaluates voluntary control of the center of gravity (COG). Instrumented LOS or FBOS, unlike the clinical Functional Reach test [8, 49], was impaired in PLHIV [7, 22]. Similarly, the Functional Reach test has been proven not to be an indicator for differentiating elderly fallers from non-fallers [70], while instrumented LOS is an early indicator of increased fall risk in the elderly [71]. These observations may be attributable to the differences in the task involved in the clinical versus the instrumented tests (although both assesses LOS) [71].

The 5STS test is an indicator of dynamic balance. Impaired performance was noted in two of the three studies evaluating this outcome [8, 9]. In addition to impaired central sensorimotor components being proposed to play a role [9], impaired 5STS time also implies poor lower limb muscle performance, which is associated with falls and disability both in HIV-seronegative elderly populations and in middle-aged PLHIV [8, 18]. Low appendicular muscle mass is prevalent in PLHIV and associated with functional impairment [72]. However, a decline in the ability of muscles to produce strength and power (dynapenia) might have a bigger contribution to functional decline in the elderly and is associated with poor chair-rise-time [73] Intra-muscular impairments, including fatty muscle infiltration, and low central activation are reported in PLHIV [29, 35, 74] and premature expression of genes associated with muscle aging is upregulated in PLHIV [75]. Grip strength might correlate with dynapenia in the elderly [76], and an accelerated decline in grip strength has been reported in PLHIV [77].

Owing to the dichotomous assessment by clinical tests of the ability to maintain standing balance, such tests only detect impaired balance once compensation strategies fail [56]. Selection of effective compensation strategies to restore balance (especially by persons who are physically active), might hide impairments, potentially hampering the use of such tests in active individuals or at an early stage of disease [56]. Level of physical activity was not assessed by any studies included in this review; ceiling effects in the results provided by the clinical balance tests can therefore not be excluded.

Although more suited to quantification of balance, interpretation of the results of instrumented posturography is complex. Generally, an increase in COP movement is assumed to reflect impaired balance; but this may not be true [78, 79]. Due to the interdependent relationship of the underlying systems, selection of an alternative compensation strategy to maintain stance could lead to observation of either increased or decreased COP movement, which in fact would reflect optimal balance control [56]. Second, altered COP movement can result from deterioration of several underlying systems. Thirdly, COP movement is affected by training and learning effects, for example, individuals (and even more so those trained in sports) might be able to maintain a position very well, despite severe system deterioration, due to becoming familiar with the task or using selecting proper strategies for efficient compensation [56]. Results, especially from singular studies, must thus be considered cautiously and in the context of the assessment protocol, e.g. number of trials, and participant characteristics, such as activity level.

### Gait

In this review, PLHIV exhibited impaired fast, but not preferred, gait speeds, despite being on successful HAART [10, 12, 22, 49]. PLHIV who were also recurrent fallers, had an even slower fast-paced gait [18]. Gait speed is reported as a predictor of falls in geriatric populations, with a linear relationship between slow gait speed and increased fall risk often assumed [80–82]. However, a non-linear relation has also been proposed [38]. Growing evidence show that gait and cognition, specifically attention and executive function [83] are interrelated. Neurocognitive decline occur in HIV [6, 13, 84–86], is in part associated with reduced dopaminergic function [87], and has been associated with slow gait speeds in this population [88]. Executive function, motor skills and attention/working memory are some of the domains that are affected by HIV [89]. Gait slowing is suggested to be an adaptive mechanism to prevent falls, to the effect that a slow gait speed might actually be associated with a reduced fall risk [38].

Six-Minute Walk Distance, which is actually an indicator of functional aerobic capacity, has been shown to correlate well with gait speed [90]. Meta-analyses of two studies [44, 49] suggests decreased 6MWD, and thus decreased gait speed under fast conditions, in PLHIV. Six-Minute Walk Distance was also reported to be decreased in PLHIV in the majority of un-pooled studies assessing this outcome [8, 9, 35] – however all of these studies used predicted values from the literature. This is of concern, as community-specific or cultural factors influence gait speed [43]. Gait initiation time was reported to be significantly slowed in PLHIV, albeit data from a single study [22]. Gait initiation time has been associated with increased fall risk in the elderly [52]. Cadence, which also has an association with gait speed and falls in the elderly [91], was assessed by two studies [7, 22], but owing to contradicting results, no firm conclusions can be drawn.

### Measurement conditions and task difficulty

Evaluating performance under conditions of varying difficulty can provide more “real-life” insight into the quality of the specific underlying sensory systems [56, 92]. Studies assessing balance included in this review employed different sensory conditions, eliminating or disturbing the information of three main sensory systems. These included variations in visual input, different base-of support sizes and variations in support surfaces. For dynamic balance assessments, perturbations of varying amplitudes and predictability were induced using platform tilts. There was an overall tendency of PLHIV to perform similar to controls in easier conditions, and significantly worse with increased task difficulty. A correlation between static balance deficit and eyes closed conditions was demonstrated by clinical as well as instrumented tests [7, 22, 48]. Unstable conditions with eyes closed showed the greatest abnormalities in postural balance [45, 46]. Sullivan et al. (2011) [21] found impaired performance among PLHIV during clinical tests involving reduced base of support. Postural reflex synergies also appear to be task-dependent. Unpredictable perturbations resulted in worse dynamic balance performance [50]. It thus seems that PLHIV may have impaired response to unexpected perturbations or more complex tasks, predisposing them to falls. In the case of impairment of any of the systems contributing to postural balance, alternative compensation strategies are used by an individual to maintain postural control and orientation [56, 93]. Sensory reweighting comes into play, i.e. the nervous system will rely on more accurate sensory input, rather than less accurate, conflicting information [94]. Individuals relying more on one particular balance system are thus less able to adapt to situations where input to that system is disturbed, and have to rely on only the remaining systems [56]. This sensory reweighting seems impaired in PLHIV. Also, impaired dual-task performance has been demonstrated in PLHIV [95], although none of the included studies assessed this condition.

All gait tests in included studies were conducted on level, unobstructed walkways. During walking, many falls occur not during normal walking, but rather when negotiation challenging terrains. Results might have been more clinically relevant had irregular or unfamiliar surfaces been assessed, especially since dynamic balance in PLHIV seem to be more impaired under challenging circumstances. However, both self-selected and fast gait speed conditions were evaluated, with group differences mostly found when comparing fast gait. It has been suggested that walking at different speeds likely influences both the noise level in human motor performance as well as dynamic error corrections [96]. Thus, impairments at fast paced conditions may indicate deficits under more challenging conditions.

### Disease severity

A dose–response relationship between HIV disease severity and locomotor impairment was suggested in 53% of studies. In addition, subgroup analyses highlighted impairments in postural reflex latencies that were inconspicuous in a combined group of all PLHIV, but became apparent in only those with symptoms of chronic HIV disease when compared to controls. However, methodologically it remains a challenge to attribute observed differences between PLHIV and controls directly to HIV infection, as evident from the discussion thus far. Comparison populations most likely always differ in terms of many confounding factors [8]. It cannot be assumed with certainty that observed impairments are purely related to severity of HIV infection, and the contribution of various comorbidities and opportunistic infections should be considered. This is especially true for the older studies, where eligibility criteria did not control for various confounders and comorbidities.

### Treatment effects: Antiretroviral therapy (ART), combination antiretroviral therapy (cART) or highly active antiretroviral therapy (HAART)

The majority of studies reporting on treatment effect failed to find significant associations to balance or gait outcomes in PLHIV, regardless of the different combinations of drugs (terms used for combination use of ARV including ART, cART or HAART). Thus, antiretroviral therapy does not appear to be a reversing factor with regard to locomotor impairments.

### Peripheral neuropathy

None of the five studies reporting on the association between peripheral neuropathy and locomotor impairments found any significant relationships. A possible explanation for balance abnormalities among PLHIV, at least for those parameters measured by the included studies, might thus indicate involvement of the central rather than peripheral nervous system [7, 8, 21, 46, 47]. The fact that eyes closed conditions were often necessary to elicit group differences in balance, further motivates CNS dysfunction as an underlying mechanism [7]. It is reported that deficits in infratentorial brain tissue volume and disruption of the pontocerebellar fiber system microstructure, at least in part, may contribute to locomotor impairments in PLHIV [21]. It can, however, not be concluded with certainty that no association exists between gait or balance and peripheral neuropathy in PLHIV. It is possible that peripheral neuropathy adversely affects gait and balance parameters that were not measured in these studies. For example, impairments in joint kinematics (assessed by none of the included studies in this review) have been associated with peripheral neuropathy in Type 2 diabetic patients [97].

## Implications for future research

While the importance of identifying spatiotemporal deficits is acknowledged, the associated kinematic and kinetic data can provide more insight into underlying mechanisms of the observed impairments. Some locomotor impairments related to early functional decline might be too small to be detected by visual observation alone in the clinical setting [51, 98]. These subtle impairments may however have substantial consequences for the individual. Thus, there is a need for more robust quantitative assessment, such as three-dimensional biomechanical motion analysis. We also suggest the use of dual tasking in PLHIV to assess the subtler changes in function, and adding more challenging conditions to gait assessments. Furthermore, a need exists for higher quality research. Carefully selected, representative samples will make results more homogeneous, relevant and generalizable. In addition, valuable information can be extracted from the geriatric literature that is likely to inform research in PLHIV, especially with regards to data on falls and specific movement impairments. This should be further explored, and the psychometric properties of both instrumented and clinical gait and balance assessments should be determined specifically in PLHIV. Lastly, we found the lack of studies conducted in Sub-Saharan Africa, the epicenter of the HIV epidemic, surprizing. More research is needed in developing countries to address this gap.

## Review limitations

Language bias is likely in this review, as only studies published in English were considered. Another limitation of the review is that only two included articles were appraised by more than one reviewer, meaning that fifteen of the seventeen articles were scored for methodological quality by only one reviewer. In addition, ceiling effects might have hampered results from clinical tests. No studies in this review measured COM movement, with a subsequent incomplete representation of balance in PLHIV at present. Results of this review should be interpreted with caution as substantial statistical heterogeneity existed between the included studies, albeit expected, as evident in the meta-analyses (indicated by high I^2^ values). Due to the small number of studies per outcome, all sources of heterogeneity could not be accounted for, but some possible explanations for variation in results have been discussed. Clinical heterogeneity was evident in the majority of studies, particularly in terms of setting, sample sizes, age groups, gender distributions, and HIV-staging. A wide variety of study outcomes and measurement methods were used. Given the paucity of research on existing impairments and the optimal method of evaluating these in PLHIV, the wide variation in assessment tests used was to be expected. Although the diversity in populations, especially regarding disease definition and subgroups, might seem surprising, it must be kept in mind that HIV classification systems have evolved substantially since the earliest included study and that HAART regimes did not yet exist in those earlier periods. Furthermore, publication- and reporting biases are suspected in this review, due to many authors collaborating on different papers and the same populations possibly used in different studies. However, formal assessment using funnel plots was not possible due to the low number (<10) studies assessing a similar outcome.

## Conclusions

This review found that young to middle-aged PLHIV have impairments in certain aspects of gait and balance, which are similar to those that predispose elderly seronegative populations to falls. The impairments are more pronounced during challenging conditions, might be associated with HIV disease severity, are not influenced by ART, and might not necessarily be associated with peripheral neuropathy. Results should be interpreted with caution due to the overall fair to low methodological quality, substantial heterogeneity and many outcomes being assessed by singular studies only. The effect of HIV on gait and balance parameters, and in particular biomechanical outcomes, are currently insufficiently quantified and this review provides a first step to inform future research. Further investigation involving more methodological uniformity is warranted to better identify and understand relevant locomotor impairments in PLHIV. Only then can more clinically relevant conclusions, such as appropriate strategies for fall-prevention in this population, be drawn.

## Additional files

Additional file 1: Results for objective balance outcomes in PLHIV (un-pooled dependent variables). Detailed summary of individual balance outcomes assessed across studies (DOCX 53 kb)

Additional file 2: Results for objective gait outcomes in PLHIV (un-pooled dependent variables). Detailed summary of individual gait outcomes assessed across studies (DOCX 101 kb)

## Abbreviations

5STS: 5-Times-Sit-To-Stand
6MWD: Six-Minute Walk Distance
AIDS: Acquired Immune Deficiency Syndrome
AP: Average velocity in an anterior-posterior direction
ART: Antiretroviral therapy
ASX: Asymptomatic HIV infection (clinically latent phase of HIV)
BMI: Body Mass Index
cART: Combination antiretroviral therapy
CD: Cannot determine
CDC: Centre for Disease Control
CI: Confidence interval
CNS: Central nervous system
COG: Center of gravity
COM: Center of mass
COP: Center of pressure
EC: Eyes closed
EMG: Electromyography
EO: Eyes open
EQ: Equilibrium Quotient
F: Female
FBOS: Functional Base of Support
FFP: Fried’s Frailty Phenotype
GIT: Gait initiation time
HAART: Highly active antiretroviral therapy
HIV: Human Immunodeficiency Virus
HRQOL: Health-related quality of life
JB: Jochen Baumeister
KB: Karina Berner
LAT: Average velocity in a medial-lateral direction
LL: Long loop
LM: Linzette Morris
LOS: Limits of Stability
M: Male
MeSH: Medical Subject Heading
ML: Medium loop
ms: Milliseconds
N: Number of participants
NA: Not applicable
NIH: National Institutes of Health
NNRTI: Non-Nucleoside Reverse Transcriptase Inhibitor
NR: Not reported
NRTI: Nucleoside Reverse Transcriptase Inhibitors
NRx: No treatment
PI: Protease Inhibitor
PLHIV: People living with HIV
PRISMA: Preferred Reporting Items for Systematic Reviews and Meta-Analyses
QL: Quinette Louw
RA: Romberg ratio of area
SD: Standard deviation
SL: Short loop
SOT: Sensory Organisation Test
SPPB: Short Physical Performance Battery
SX: Symptomatic (presenting with various symptoms of chronic HIV disease)
TUG: Timed Up-And-Go
WR: Walter Reed staging
