# Supplementary material for: Objective impairments of gait and balance in adults living with HIV-1 infection: a systematic review and meta-analysis of observational studies
Source: BMC Musculoskelet Disord. 2017 Aug 1;18:325. doi: 10.1186/s12891-017-1682-2 (PMC5540197; doi:10.1186/s12891-017-1682-2)
Supplement: Supplementary file 1 — Results for objective balance outcomes in PLHIV (un-pooled dependent variables). Detailed summary of individual balance outcomes assessed across studies (DOCX 53 kb) [file 12891_2017_1682_MOESM1_ESM.docx]

**Additional file 1** Results for objective balance outcomes in PLHIV (un-pooled dependent variables)

| **Study ID** | **Method of assessment** | **Results of dependent variables** | | **Summary of findings** |
| --- | --- | --- | --- | --- |
|  |  | **PLHIV** | **CONTROL** |  |
| **Mean sway path: m/min (SD)** | | | | |
| **Trenkwalder 1992** [46] | 4 conditions on force plate:  (1) Bilateral stance, EO, firm support  (2) Bilateral stance, EC, firm support  (3) Bilateral stance, EO, foam  (4) Bilateral stance, EC, foam | WR I-II  EO, firm: 0.40 (0.12)  EC, firm: 0.75 (0.43)  EO, foam: 0.93 (0.40)  EC, foam: 3.46 (1.40)*  WR III-V  EO, firm: 0.44 (0.15)  EC, firm: 0.79 (0.35)  EO, foam: 1.04 (0.65)*  EC, foam: 3.37 (0.78)*  WR VI  EO, firm: 0.65 (0.40)  EC, firm: 0.81 (0.36)  EO, foam: 1.02 (0.37)*  EC, foam: 3.70 (1.06)* | EO, firm: 0.43 (0.10)  EC, firm: 0.79 (0.32)  EO, foam: 0.63 (0.17)  EC, foam: 2.60 (0.72) | - Significant instability in PHIV when standing on unstable support. - All HIV groups had significantly higher postural sway in EC condition versus controls, regardless of support. |
| **Sway velocity: m/sec (SD)** | | | | |
| **Arendt** **1994** [47] | 2 conditions on force plate:  (1) Bilateral stance, EO  (2) Bilateral stance, EC | ASX  EO: 0.88 (0.34)  EC: 1.23 (0.60)  SX  EO: 1.75 (0.85)*  EC: 3.12 (0.5)* | EO: 0.78 (0.20)  EC: 1.12 (0.35) | - Significant increase in sway velocity in neurologically SX PLHIV for EO as well as EC conditions. - Increase in sway velocity in approximately 25% of ASX PLHIV (although clinical relevance discounted by authors). |
| **Average velocity in anterior-posterior direction; AP (SD)** | | | | |
| **Dellepiane 2005** [48] | 2 conditions on force plate:  (1) Bilateral stance, EO  (2) Bilateral stance, EC | ASX EO: 0.14 (0.08)  EC: 0.35 (0.30)* SX EO: 0.22 (0.07)* EC: 0.39 (0.23)* | EO: 0.12 (0.07) EC: 0.16 (0.09) | - ASX PHIV: Significantly increased in EC (p < 0.05) versus controls. - SX PLHIV: Significantly increased both in EO and EC (p < 0.05). |
| **Average velocity in medial-lateral direction; LAT (SD)** | | | | |
| **Dellepiane 2005** [48] | 2 conditions on force plate:  (1) Bilateral stance, EO  (2) Bilateral stance, EC | ASX EO: 0.18 (0.07)*  EC: 0.32 (0.14)  SX  EO: 0.22 (0.13)  EC: 0.46 (0.39)* | EO: 0.24 (0.01) EC: 0.37 (0.15) | - ASX PLHIV: Significantly decreased in EO (p < 0.05) versus controls. - SX PLHIV: Significantly increased in EC (p 0.05) but not in EO. |
| **AP/LAT quotient** | | | | |
| **Arendt 1994** [47] | 2 conditions on force plate:  (1) Bilateral stance, EO  (2) Bilateral stance, EC | ASX  EO: 3.11/2.46 = 1.26  EC: 3.5/2.25 = 1.56  SX  EO: 2.5/1.5 = 1.67  EC: 4.38/3.15 = 1.39 | EO: 2.13/2.59 = 0.82  EC: 4.51/3.74 = 1.21 | - PLHIV similar to controls, with predominance of AP over LAT sway components. |
| **Dellepiane 2005** [48] | 2 conditions on force plate:  (1) Bilateral stance, EO  (2) Bilateral stance, EC | ASX EO: 0.88 (0.50) EC: 0.55 (0.14)  SX EO: 1.13 (0.41)  EC: 1.38 (1.35) | EO: 0.60 (0.35) EC: 0.55 (0.41) | - Predominance of AP over LAT sway components on HIV as well as control groups. |
| **Romberg ratio of area; RA (SD)** | | | | |
| **Dellepiane 2005** [48] | 2 conditions on force plate:  (1) Bilateral stance, EO  (2) Bilateral stance, EC | ASX: 2.98 (1.81)* SX: 2.43 (1.17)* | 1.94 (0.91) | - Significantly increased in all the PLHIV groups versus controls (p < 0.05). |
| **Latencies of postural reflexes: ms (SD)** | | | | |
| **Dellepiane 2005** [48] | 2 conditions on movable force plate with sudden tilts; surface EMG recorded from triceps sural and tibialis anterior muscles bilaterally: (1) Bilateral stance, EO (2) Bilateral stance, EC | ASX, EO ML, right: 60.0 (11.8) ASX, EC SL, right: 43.6 (3.7)*  SL, left: 47.3 (8.3)*  ML, right: 65.3 (11.8)  ML, left: 68.6 (5.5)*  LL, right: 122.6 (13.4)  LL, left: 114.3 (10.2)  SX, EO  ML, right: 67.2 (3.9)*  SX, EC SL, right: 42.2 (0.5)  SL, left: 46.5 (1.1)  ML, right: 70.7 (6.1)  ML, left: 55.6 (4.2)  LL, right: 144.2 (70.7)*  LL, left: 143.2 (58.7) * | EO ML, right: 61.9 (3.7) EC SL, right: 39.5 (3.1)  SL, left: 40.8 (3.5)  ML, right: 63.3 (6.8)  ML, left: 63.0 (4.9)  LL, right: 113. (19.4)  LL, left: 118.0 (18.7) | - In both EO and EC, all PLHIV showed an increase of SL latency compared to controls (p < 0.05). - With EO, ML latency showed an increase in the SX PLHIV both on the left and right side, and only on the left side in the ASX group (p < 0.05). - LL latency was increased (p < 0.05) only in the SX group with EC. - With EC, no significant differences in ML latency were observed in all PLHIV. |
| **Duration of postural reflexes** | | | | |
| **Dellepiane 2005** [48] | 2 conditions on movable force plate with sudden tilts; surface EMG recorded from triceps sural and tibialis anterior muscles bilaterally: (1) Bilateral stance, EO (2) Bilateral stance, EC | ASX, EO SL, right: 19.7 (0.9)  SL, left: 20.5 (2.8)  ML, right: 29.5 (24.0)  ML, left: 18.5 (7.0)  LL, right: 182.0 (34.0)* LL, left: 180.0 (44.4)* ASX, EC SL, right: 20.0 (3.0)  SL, left: 19.3 (3.0)  ML, right: 19.6 (1.5)* ML, left: 14.6 (3.2)* LL, right: 136.6 (18.9)* LL, left: 168.3 (17.6)* SX, EO SL, right: 19.6 (5.0) SL, left: 17.4 (5.4)  ML, right: 18.6 (4.4)  ML, left: 20.0 (8.4)  LL, right: 183.5 (22.5)* LL, left: 190.4 (46.1)* SX, EC SL, right: 22.7 (5.8)  SL, left: 16.7 (2.8)* ML, right: 13.0 (6.7)* ML, left: 12.6 (6.5)* LL, right: 135.4 (21.1)* LL, left: 119.0 (30.1)* | EO SL, right: 19.7 (2.9) SL, left: 20.8 (3.7)  ML, right: 21.6 (4.9)  ML, left: 21.0 (4.5) LL, right: 128.8 (48.2)  LL, left: 130.2 (42.7)  EC SL, right: 21.6 (3.6)  SL, left: 20.7 (3.7)  ML, right: 26.9 (5.4)  ML, left: 27.5 (6.4)  LL, right: 175.2 (54.9)  LL, left: 167.3 (53.0) | - No significant difference in the duration of SL and ML with EO in ASX or SX PLHIV versus controls. - With EC, there was a significant reduction (p < 0.05) of SL to the left in the SX group and of ML bilaterally in all PLHIV. - LL duration was increased with EO and reduced with EC (p < 0.05). |
| **Amplitude of postural reflexes** | | | | |
| **Dellepiane 2005** [48] | 2 conditions on movable force plate with sudden tilts; surface EMG recorded from triceps sural and tibialis anterior muscles bilaterally: (1) Bilateral stance, EO (2) Bilateral stance, EC | ASX, EO SL, right: 361 (276) SL, left: 361 (279) ML, right: 234 (125) ML, left: 290 (232) LL, right: 590 (158) LL, left: 785 (200)  ASX, EC SL, right: 446 (169) SL, left: 568 (154) ML, right: 204 (157) ML, left: 400 (277) LL, right: 655 (180) LL, left: 670 (144)  SX, EO SL, right: 398 (152) SL, left: 464 (171) ML, right: 280 (100) ML, left: 374 (165) LL, right: 587 (160) LL, left: 610 (205) SX, EC SL, right: 480 (65) SL, left: 563 (73) ML, right: 332 (164) ML, left: 363 (208) LL, right: 609 (261) LL, left: 479 (184) | EO SL, right: 406 (264) SL, left: 469 (238) ML, right: 338 (239) ML, left: 317 (211) LL, right: 732 (236) LL, left: 666 (201  EC SL, right: 452 (196) SL, left: 384 (247) ML, right: 339 (172) ML, left: 319 (195) LL, right: 689 (145) LL, left: 674 (398) | - No significant differences between PLHIV and controls. |
| **Area of single EMG potential** | | | | |
| **Dellepiane 2005** [48] | 2 conditions on movable force plate with sudden tilts; surface EMG recorded from triceps sural and tibialis anterior muscles bilaterally: (1) Bilateral stance, EO (2) Bilateral stance, EC | ASX, EO SL, right: 2.8 (0.6) SL, left: 2.1 (1.0) ML, right: 3.9 (2.9)  ML, left: 4.3 (3.1)  LL, right: 43.7 (7.4) LL, left: 58.7 (9.5)  ASX, EC SL, right: 4.8 (1.8)  SL, left: 6.5 (1.8)  ML, right: 3.9 (0.5)  ML, left: 4.0 (1.6)  LL, right: 30.4 (13.9)  LL, left: 44.1 (5.0)  SX, EO SL, right: 4.5 (1.8) SL, left: 4.6 (1.9) ML, right: 3.0 (1.2) ML, left: 4.6 (3.1)  LL, right: 45.2 (12.7)  LL, left: 40.9 (16.9)  SX, EC SL, right: 5.4 (0.4)  SL, left: 5.8 (1.1)  ML, right: 5.0 (3.5)  ML, left: 5.1 (3.2)  LL, right: 29.8 (9.0)  LL, left: 30.1 (19.8) | EO SL, right: 4.3 (2.3)  SL, left: 4.8 (2.8) ML, right: 3.9 (2.3) ML, left: 3.6 (1.9) LL, right: 51.4 (29.2)  LL, left: 44.6 (21.5)  EC SL, right: 5.2 (2.0) SL, left: 4.4 (2.8) ML, right: 4.8 (0.3) ML, left: 4.5 (2.3) LL, right: 57.4 (50.5) LL, left: 48.9 (34.4) | - No significant differences between PLHIV and controls. |
| **Normalized amplitude of the ML response: ML/SL ratio (SD)** | | | | |
| **Beckley 1998** [50] | Bilateral stance on movable force plate with toe-up tilts of varying amplitude and predictability; surface EMG recorded from left medial gastrocnemius and tibialis anterior muscles | During 4º predictable condition: 1.2 (1.0) | During 4º predictable condition: 2.4 (2.1) | - Amplitudes of the ML responses are normal in PLHIV exposed to predictable postural perturbations. |
| **Standardised LL Z scores (LL-amplitude scaling)** | | | | |
| **Beckley 1998** [50] | Bilateral stance on movable force plate with toe-up tilts of varying amplitude and predictability; surface EMG recorded from left medial gastrocnemius and tibialis anterior muscles | LL amplitude scaling in 56% | LL amplitude scaling in 70% | - In both PLHIV and controls, LL responses varied appropriately with perturbation size under *predictable* test conditions. - Neurologically intact PLHIV showed abnormal regulation of postural reflexes under *unpredictable* perturbations to stability, but normal postural reflexes when responding to *predictable* task conditions. - Clinical relevance queried by authors. |
| **Way (average velocity of movement; speed of COG)** | | | | |
| **Dellepiane 2005** [48] | Static posturography: Standing in Romberg’s position on force plate; 2x conditions (EO & EC) | ASX EO: 1.23 (0.32)  EC: 2.41 (0.90)  SX EO: 1.79 (0.96)* EC: 3.21 (1.92)* | EO: 1.32 (0.41) EC: 1.93 (0.59) | - Significant increase (p<0.05) both in EO and EC conditions in SX PLHIV versus controls. |
| **SOT sway strategy score: Mean(95% CI)** | | | | |
| **Bauer 2011** [22] | Computerised SOT on force plate; 3 conditions:  (1) Bilateral stance, EO (SOT4)  (2) Bilateral stance, EC (SOT5) (3) Bilateral stance, inaccurate visual input (SOT6) | SOT 4  BMI<21:84.4 (82–87)  BMI 21-29: 83.2 (81–84)  BMI>29: 74.1 (70–76)  SOT 5  BMI<21: 75.5 (72–79)  BMI 21-29: 69.8 (65–70)  BMI>29:64.7 (62–69)  BMI>29: 57.4 (53–62)  SOT 6  BMI<21: 74.2 (12.23)  BMI21-29: 73.2(20.30)  BMI>29: 63.9(60-69) | SOT4 BMI<21: 85.4(82-88)  BMI21-29: 81.6(79-84)  BMI>29: 80.8(78-83)  SOT5 BMI<21: 78.1(73-82)  BMI21-29: 71.7(62-69)  BMI>29: 64.7(62-69)  SOT6: BMI<21: 78.0(73-83)  BMI21-29: 69.6(65-73)  BMI>29: 68.7(65-69) | - HIV was associated with a lower sway strategy score on the SOT5 subtest (F=6.1, p<0.01). - Statistically significant correlations between CD4 count and SOT5 sway strategy score: SOT5 strategy score improved with higher CD4 count (r=0.25, P=0.01). - Synergism in the statistical effects of HIV/AIDS and BMI on sway strategy scores during the SOT4 and SOT5 subtests. |
| **SOT EQ** | | | | |
| **Bauer 2005** [7] | Computerised SOT on force plate; 3 conditions:  (1) Bilateral stance, EO  (2) Bilateral stance, EC  (3) Bilateral stance, inaccurate visual input | (only EC condition reported)  Cocaine, never: 50.9 (11) Cocaine, prev: 49.4 (13) Cocaine, current: 45.9 (15) Opiod, never: 48.6 (13)  Opiod, prev: 50.4 (10)  Opiod, current: 49.3 (17) Alcohol, never: 50.7 (12) Alcohol, prev: 47.2 (14) Alcohol, current: 44.2 (7) MDD, negative: 49.3 (12) MDD, positive: 49.2 (13) | NR | - Significant differences between PLHIV and controls (p < 0.03) during EC condition only, especially during the most difficult SOT subtest (sway-referenced support surface with EC). - Authors question clinical significance. |
| **Bauer 2011** [22] | Computerised SOT on force plate; 3 conditions:  (1) Bilateral stance, EO (SOT4)  (2) Bilateral stance, EC (SOT5) (3) Bilateral stance, inaccurate visual input (SOT6) | *Mean (95% CI)*  SOT 4  BMI<21: 75.2 (71–81)  BMI 21-29: 80.3 (76–84)  BMI>29: 76.2 (70–82)  SOT 5  BMI<21:46.1 (42–51)  BMI 21-29: 50.6 (47–54)  BMI>29: 51.9 (46–57)  SOT 6  BMI<21: 47.5 (43–53)  BMI 21-29: 56.4 (52–60)  BMI>29: 55.4 (51–63) | *Mean (95% CI)*  SOT4  BMI <21: 75.6 (69–81)  BMI 21-29: 78.3 (73–83)  BMI>29:80.3 (76–85)  SOT5  BMI <21: 57.7 (51–62)  BMI 21-29: 56.3 (52–61)  BMI>29: 56.5 (52–60)  SOT6  BMI <21: 57.1 (50–63)  BMI 21-29: 57.7 (51–61)  BMI>29: 60.5 (55–65) | - Lower EQ scores in PLHIV on the SOT5 (p<0.001), and the SOT6 (p<0.02), versus controls. |
| **SOT number of falls: count; time before fall: sec** | | | | |
| Ba**uer 2005** [7] | Computerised SOT on force plate; 3 conditions:  (1) Bilateral stance, EO  (2) Bilateral stance, EC  (3) Bilateral stance, inaccurate visual input | NR | NR | - No significant group differences between PLHIV and controls. |
| **Functional Base of Support; Limits of Stability: lean amplitude divided by foot length (SD)** | | | | |
| **Bauer 2005** [7] | Forward/backward lean tests: Standing on fixed support, wearing shoulder harness. | Cocaine, never: 0.53 (0.1) Cocaine, previous: 0.52 (0.1) Cocaine, current: 0.49 (0.1) Opiod, never: 0.53 (0.1)  Opiod, previous: 0.52 (0.1)  Opiod, current: 0.46 (0.1) Alcohol, never: 0.53 (0.1) Alcohol, previous: 0.51 (0.1) Alcohol, current: 0.46 (0.1) MDD, negative: 0.52 (0.1)  MDD, positive: 0.51 (0.1) | NR | - Significant group differences (p < 0.004) between PLHIV and controls. - All PLHIV differed significantly from controls on the Limits of Stability. - CD4 count was positively related to the Functional Base of Support (p < 0.05). |
| **Bauer 2011** [22] | Limits of Stability: Forward/backward lean tests with heels kept on floor. | *Median (IQR)*  BMI<21: 0.52 (0.49–0.55)  BMI 21-29: 0.53 (0.51–0.56)  BMI>29: 0.45 (0.41–0.49) | *Median (IQR)*  BMI <21: 0.61 (0.56–0.64)  BMI 21-29: 0.56 (0.52–0.59)  BMI>29: 0.52 (0.47–0.53) | - Reduced LOS in HIV-groups (p<.001). |
| **Berg balance score** | | | | |
| **Richert 2011** [8] | Berg balance scale, assessing overall balance and equilibrium by the means of 14 functional tasks | *Median, IQR* 56 (55-56) | Score of <46 considered as poor performance in all age categories. | - Group result of PLHIV above threshold for poor performance. - Proportion of PLHIV with poor performance: 1.5% (95% CI 0.5, 3.6). |
| **TUG time: sec** | | | | |
| **Richert 2011** [8] | TUG test | *Median (IQR)*  5.6 (5.1 – 6.4) | Poor performance was defined by a result of >2SD from the expected age-specific mean in the general population. | - PLHIV similar to normative values. - Proportion of PLHIV with poor performance: 10.5% (96% CI 7.4, 14.4). |
| **Richert 2014** [9] | TUG test | *Median (IQR)*  Follow-up (n=178): 5.1 (4.7, 5.7) | NA | - In this longitudinal study, an estimated annual change in performance of mean (95% CI): -0.27 (-0.34, -0.20) is reported in PLHIV (p<10 ^-4^). - Thus, TUG time appeared to improve over time (baseline value 5.6 (5.1-6.4). The authors suggested learning effects from one study phase to the other as a possible explanation. |
| **5STS pace: rises/second (SD)** | | | | |
| **Erlandson 2014** [12] | 5STS | 0.51 (0.19) | NA | - Multivariable linear regression models found that for every increase of 1 rise/sec on chair rise pace, there was an estimated 16.0 point increase in SF-36 physical function scores and 15.0 point increase in SF-36 social function scale. - Faster chair rise time were associated with greater QOL among adults aging with effectively controlled HIV. |
| **5STS time: sec** | | | | |
| **Bauer 2011** [22] | 5STS | *Median(IQR)*  BMI<21: 10.2 (9.4–10.8)  BMI 21-29: 10.2 (9.6–10.6)  BMI>29: 10.2 (9.4–11.2) | *Median(IQR)*  BMI <21: 9.5 (8.5–10.4)  BMI 21-29: 9.8 (8.8–10.4)  BMI>29: 9.5 (8.9–10.4) | - No group differences observed. |
| **Richert 2011** [8] | 5STS | *Median(IQR)*  9.8 (8.3 – 11.4) | Results interpreted according to data established in the general population. Poor performance was defined by a result of >2SD from the expected age-specific mean. | - High frequency of poor performance in PLHIV. - Proportion of PLHIV with poor performance: 53.3% (95% CI: 47.6, 58.8). |
| **Richert 2014** [9] | 5STS | *Mean (95% CI)*  Follow-up (n=178): 10.3 (9.0, 12.2)  Estimated annual change: 0.24 (0.07, 0.42) | NA | - Mean deterioration was +0.24 sec/year (95% CI 0.07, 0.42; P < 10 -2). - 31% of participants had a worsening in 5STS time >empirically defined threshold of 2 sec. - At baseline, participants had an average 5STS time of 9.8 sec. In healthy persons of similar age, performance of 7.1 sec has been reported. |
| **Romberg ECF (seconds)** | | | | |
| C**ohen 2012** [45] | Romberg standing balance test on foam measuring failure to maintain balance during ECF for 30s | NR | NR | - Frequency of impairment slightly higher in PLHIV than controls (15.6% and 13.9%, respectively). |
| **360 degree turn time: sec (IQR)** | | | | |
| **Bauer 2011** [22] | 360-degree turn test | BMI<21: 1.5 (1.4–1.6)  BMI 21-29: 1.5 (1.4–1.6)  BMI>29:2.3 (2.1–2.5) | BMI <21: 1.4 (1.2–1.6)  BMI 21-29:1.7 (1.5–1.8)  BMI>29: 1.7 (1.6–1.8) | - 360-degree turn time improved with higher CD4 counts (p=0.02). - Synergism observed in the statistical effects of HIV/AIDS and BMI. - No effect of PI treatment. |
| **Tandem stance: sec (SE)** | | | | |
| **Sullivan 2011** [21] | Walk-a-Line Battery of gait and balance = 3 tasks, each performed with EO and EC, arms folded. | EO  M: 109.6 (5.72)  F: 97.7 (9.92)  EC  M: 65.0 (8.32)  F: 48.7 (12.26) | EO  M: 118.2 (1.15)  F: 109.3 (3.63)  EC  M: 69.9 (7.11)  F: 63.3 (7.3) | - PLHIV was not impaired on the Romberg test (standing heel-to-toe) assessing proprioception (p-values not meeting conservative significance level of ≤0.01). |
| **Walk heel-to-toe: number of steps (SE)** | | | | |
| **Sullivan 2011** [21] | Walk-a-Line Battery of gait and balance = 3 tasks, each performed with EO and EC, arms folded. | EO  M: 16.9 (1.14)  F: 14.5 (1.91)  EC  M: 3.9 (0.50)*  F: 3.1 (0.56)* | EO  M: 18.7 (0.58)  F: 17.5 (0.74)  EC  M: 5.8 (0.66)  F: 5.3 (0.67) | - Significant group differences in heel-to-toe walking in EC condition between PLHIV and controls (p=0.0066). |
| **Forward reach distance: cm** | | | | |
| **Simmonds 2005** [49] | (1) Patients stood adjacent to a wall on which a horizontal measure was positioned at shoulder height. They reached forward as far as possible.  (2) Second condition as above, but holding a weight at shoulder height. | *Mean (SD)*  Unloaded: 34.54 (7.32)  Loaded: 57.36 (12.33) | *Mean (SD)*  Unloaded: 34.76 (8.09)  Loaded: NR | - Unloaded forward reach distance similar in PHIV versus controls. |
| **Richert 2011** [8] | Functional reach test: maximal distance that a participant can reach forward beyond arm’s length while maintaining a fixed base of support in the standing position. | *Mean (IQR)*  36 (31–43) | Results interpreted according to data established in the general population. Poor performance was defined by a result of >2SD from the expected age-specific mean. | - Proportion of PLHIV with poor performance: 10.6% (95% CI 7.4, 14.4). |
| **SLST time: sec** | | | | |
| **Richert 2011** [8] | SLST, EC | *Median (IQR)*  12. 6 (6.8 – 24.0) | Results were interpreted according to data established in the general population. Poor performance was defined by a result of >2SD from the expected age-specific mean. | - Proportion of PLHIV with poor performance: 9.9% (95% CI 6.9, 13.7). |
| **Bauer 2011** [22] | SLST, preferred and unpreferred leg | *Mean (95% CI)*  Nonpreferred  BMI<21: 25.5 (23–28)  BMI 21-29: 25.2 (23–27)  BMI>29: 18.4 (15–21)*  Preferred  BMI<21: 23.7 (21–26)  BMI 21-29: 26.3 (24–27)  BMI>29: 21.6 (17–23) | *Mean (95% CI)*  Nonpreferred  BMI <21: 27.4 (24–30)  BMI 21-29: 24.0 (22–27)  BMI>29: 23.6 (20–26)  Preferred  BMI <21: 27.8 (24–30)  BMI 21-29: 24.3 (22–27)  BMI>29: 24.1 (21–26) | - Synergistic interaction of serostatus and BMI: PLHIV with an obese BMI were impaired relative to those with an underweight BMI and the other participant groups in nonpreferred leg stance time (p<0.04). |
| **Sullivan 2011** [21] | SLST on left as well as right foot, EO and EC (part of Walk-a-Line Battery of Gait and Balance) | *Mean (SE)*  Left, EO*  M: 49.6 (3.86)*  F: 41.8 (6.51)*  Right, EO  M: 54.3 (2.59)  F: 51.5 (4.70)  Left, EC*  M: 12.6 (1.46)*  F: 17.9 (4.93)*  Right, EC  M: 17.8 (3.20)  F: 24.5 (6.61) | *Mean (SE)*  Left, EO  M: 55.9(1.96)  F: 54.7 (2.13)  Right, EO  M: 58.3 (1.17)  F: 55.5 (1.87)  Left, EC  M: 28.9 (3.41)  F: 22.6 (3.15)  Right, EC  M: 30.5 (3.19)  F: 24.7 (3.25) | - PLHIV exhibited performance deficits in balancing on one foot. - Significant differences between PLHIV and controls were forthcoming on standing on the left foot, both EO (p=0.0045) and EC (p=0.0052) (predominantly right-handed participant-group). |
| **Richert 2014** [9] | SLST, EC | *Median (IQR)*  16.0 (7.2, 30.0) | No control / comparison with normative values. | - In this longitudinal follow-up study, the SLST (EC) appeared to improve over time (baseline value 12.7 (7.0, 25.0). The authors suggested learning effects from one study phase to the other as a possible explanation. |
| Outcomes pooled in meta-analyses are not included in this narrative summary.  *statistically significant difference versus controls; all results reported as mean (SD) unless otherwise specified.  *Abbreviations: AIDS* acquired immunodeficiency syndrome*, ASX* asymptomatic*, BMI* body mass index*, CI* confidence interval*, EC* eyes closed*, ECF* eyes-closed-on-foam*, EO* eyes open*, F* females*, HIV* human immunodeficiency virus, *IQR* interquartile range, *LL* long loop*, M* males*, m* meters*, MDD* major depressive disorder*, min* minute*, ML* medium loop*, ms* milliseconds*, NA* not applicable, *NR* not reported, *PLHIV* people living with HIV*, QOL* quality of life, *SD* standard deviation*, sec* second*, SL* short loop*, SLST* single leg stance test*, SOT* sensory organization test, *SX* symptomatic*,* TUG timed-up-and-go, *WR* Walter Reed staging. | | | | |
