# Supplementary material for: Objective impairments of gait and balance in adults living with HIV-1 infection: a systematic review and meta-analysis of observational studies
Source: BMC Musculoskelet Disord. 2017 Aug 1;18:325. doi: 10.1186/s12891-017-1682-2 (PMC5540197; doi:10.1186/s12891-017-1682-2)
Supplement: Supplementary file 2 — Results for objective gait outcomes in PLHIV (un-pooled dependent variables). Detailed summary of individual gait outcomes assessed across studies (DOCX 101 kb) [file 12891_2017_1682_MOESM2_ESM.docx]

**Additional file 2** Results for objective gait outcomes in PLHIV (un-pooled dependent variables)

| **Study ID** | **Method of assessment** | **Results of dependent variables** | | **Summary of findings** |
| --- | --- | --- | --- | --- |
|  |  | **PLHIV** | **CONTROL** |  |
| **Gait speed: m/sec** | | | | |
| **Bauer 2011** [22] | 8-m walk (preferred and fast pace) recorded with photocells and automatic timers | *Mean (95% CI)*  Preferred  BMI<21: 1.22 (1.1–1.3)  BMI 21-29: 1.24 (1.2–1.3)  BMI>29: 1.18 (1.0–1.3)  Fast  BMI<21: 1.94 (1.8–2.0)  BMI 21-29: 2.01 (1.9–2.1)  BMI>29: 1.79 (1.6–1.9) | *Mean (95% CI)*  Preferred  BMI <21: 1.32 (1.2–1.4)  BMI 21-29: 1.25 (1.2–1.4)  BMI>29: 1.16 (1.1–1.2)  Fast  BMI <21: 2.15 (2.0–2.3)  BMI 21-29: 2.04 (1.9–2.2)  BMI>29: 1.99 (1.8–2.0) | - HIV was associated with reduced fast gait speed (p<0.002). |
| **Erlandson 2012a** [10] | 400-m walk (fast pace) | *Mean (SD)*  Fast  High Fx: 1.7(0.1)  Moderate Fx: 1.3(0.2)  Low Fx: could not complete | NA | - More than 50% of middle-aged PLHIV on successful ART were unable to maintain a fast gait speed faster than 3.4 miles/h (5.5 km/h; 1.5m/sec) in the 400-m walk test, thus meeting a criterion for Social Security Administration disability, and an additional 3% of PLHIV were unable to walk a distance of 400-m at any pace. - By 400-m walk, 46% were high-, 51% moderate-, and 3% low-function. |
| **Erlandson 2012b** [18] | 400-m walk (fast pace) | *Mean (SE)*  Fast  Non-fallers: 1.52 (0.02)  Recurrent fallers: 1.33 (0.04) | NA | - PLHIV who were recurrent fallers had a significantly slower pace on the 400-m walk than non-fallers (p <0.001). |
| **Richert 2014** [9] | 10-m walk (pace not reported) | *Mean (95% CI)*  Baseline: 1.9 (1.7, 2.1)  Follow-up: 1.9 (1.8, 2.2) Estimated annual change: 0.04 (0.02, 0.05) | NA | - No deterioration after 2 year follow-up was found in this longitudinal study (*p<10 ^-4^* ). |
| **Erlandson 2014** [12] | 400-m walk (fast pace) | *Mean (SD)*  1.43 (0.37) | NA | - 3% were unable to complete the 400-m walk. - Faster gait speed was associated with greater QOL. - For every 1 m/sec increase in gait speed, there was an estimated 11.8-point mean increase in the SF-36 physical function subscale and an 8.4-point mean increase in the role physical subscale. |
| **Timed gait: sec** | | | | |
| **Bauer 2005** [7] | 8-m walk (preferred and fast pace) | NR | NR | - No significant differences between PLHIV and controls. |
| **Simmonds 2005** [49] | 15.24-m (50-foot) walk (preferred and fast pace) | *Mean (SD)*  Preferred: 15.36 (3.17)  Fast: 11.36 (2.53)* | *Mean (SD)*  Preferred: Not reported  Fast: 8.87 (1.93) | - PLHIV had significantly decreased fast 15.24-m pace versus controls. |
| **Beans 2013** [43] | 400-m long distance corridor walk | *Mean (SE)*  258.5 (4.9) | *Mean (SE)*  276.3 (5.5) | - Time to complete the 400-meter walk was shorter in PLHIV versus controls (p=0.02), indicating better performance. - Authors questioned clinical significance. |
| **6MWD: m^a^** | | | | |
| **Scott 2007** [35] | 6MWT | *Mean (SD)*  593 (73) | No control group – used predicted values, based on age and BMI, for comparison. | - PLHIV appeared to have decreased 6MWD, compare to values predicted based on BMI and age. |
| **Richert 2011** [8] | 6MWT | *Mean (SD)*  554 (83) | Results interpreted according to data established in the general population. | - Poor performance in a quarter of PLHIV, based on normative data, generated by Enright’s reference equation using age, sex, weight, and height. |
| **Beans 2013** [43] | 6MWT | *Mean (SE)*  545 (10) | *Mean (SE)*  531 (11) | - No significant difference between PLHIV and controls (p=0.3). |
| **Richert 2014** [9] | 6MWT | *Median(IQR)*  Baseline: 548 (500, 613)  Follow-up: 520 (480, 575)  *Mean (95% CI)*  Estimated annual change:-11 (-16, -6) | Predicted values according to literature. | - Estimated mean deterioration was -11 m/year (p < 10^-4^) for 6MWD. - 34% of PLHIV had a decline in 6MWD of at least -54 m over 2 years, which corresponds to the minimal important difference in patients with cardiopulmonary disease. - Results show that 6MWD is impaired (an average of approximately 640m would have been expected according to a published reference formula for healthy individuals of the same age, sex, and BMI) and further declines over time in PLHIV (a mean annual decline of approximately -5 m could have been expected in the general population). |
| **Cadence: time in sec for 5 steps** | | | | |
| **Bauer 2005** [7] | 8-m walk (preferred and fast pace) | NR | NR | - No significant differences between PLHIV and controls. |
| **Bauer 2011** [22] | 8-m walk (preferred and fast pace) (recorded with photocells and automatic timers) | *Mean (95% CI)*  Preferred  BMI<21: 5.33 (5.2–5.5)  BMI 21-29: 5.30 (5.2–5.5)  BMI>29: 5.40 (5.2–5.6)  Fast  BMI<21: 4.01 (3.8–4.1)  BMI 21-29: 3.96 (3.8–4.1)  BMI>29: 4.24 (4.0–4.4) | *Mean (95% CI)*  Preferred  HIV-, BMI <21: 5.22 (5.0–5.4)  BMI 21-29: 5.53 (5.3–5.7)  BMI>29: 5.54 (5.3–5.7)  Fast  BMI <21: 3.82 (3.6–4.0)  BMI 21-29: 4.08 (3.9–4.2)  BMI>29 kg/m2: 3.97 (3.8–4.2) | - Synergistic interaction of HIV and BMI. PLHIV who were also obese were impaired relative to those with an underweight body mass and the other participant groups in fast gait cadence time (P<0.05). |
| **Gait initiation time, preferred and fast: sec** | | | | |
| **Bauer 2011** [22] | 8-m walk, preferred and fast pace (recorded with photocells and automatic timers) | *Mean (95% CI)*  Preferred  BMI<21: 0.59 (0.56–0.62)  BMI 21-29: 0.57 (0.54–0.59)  HIV+, BMI>29kg/m2: 0.61 (0.56–0.64)  Fast  BMI<21:0.40 (0.38–0.42)  BMI 21-29: 0.39 (0.37–0.40)  BMI>29: 0.48 (0.46–0.50) | *Mean (95% CI)*  Preferred: BMI <21: 0.54 (0.49–0.57)  BMI 21-29: 0.54 (0.51–0.58)  BMI>29: 0.59 (0.55–0.62)  Fast:  BMI <21: 0.36 (0.34–0.39)  BMI 21-29: 0.37 (0.35–0.39)  BMI>29: 0.38 (0.37–0.41) | - HIV was associated with delayed normal gait initiation time (p<0.03). - Synergistic interaction of HIV and BMI. Participants who were HIV+ and obese were impaired relative to those with an underweight body mass and the other participant groups in fast gait initiation time (p<0.05). |
| Outcomes pooled in meta-analyses are not included in this narrative summary.  *statistically significant difference versus controls; all results reported as mean (SD) unless otherwise specified.  *Abbreviations: 6MWT* 6-minute walk distance*, 6MWT* 6-minute walk test*, AIDS* acquired immunodeficiency syndrome*, ASX* asymptomatic*, BMI* body mass index*, CI* confidence interval*, EC* eyes closed*, EO* eyes open*, F* females*, HIV* human immunodeficiency virus, *IQR* interquartile range*, min* minute*, ms* milliseconds*, NA* not applicable, *NR* not reported, *PLHIV* people living with HIV*, QOL* quality of life, *SD* standard deviation*, sec* second*, SF-36* Short Form 36 Health Survey*, SX* symptomatic*, WR* Walter Reed staging. | | | | |
